# Histone Methylation Is Required for Virulence, Conidiation, and Multi-Stress Resistance of *Alternaria alternata*

**DOI:** 10.3389/fmicb.2022.924476

**Published:** 2022-06-16

**Authors:** Shuai Meng, Suya Huang, Jinhua Liu, Yunpeng Gai, Min Li, Shuo Duan, Shuting Zhang, Xuepeng Sun, Qi Yang, Yuchun Wang, Kai Xu, Haijie Ma

**Affiliations:** ^1^Collaborative Innovation Center for Efficient and Green Production of Agriculture in Mountainous Areas of Zhejiang Province, College of Horticulture Science, Zhejiang A&F University, Hangzhou, China; ^2^Natural Medicine Institute of Zhejiang YangShengTang Co., LTD, Hangzhou, China; ^3^School of Grassland Science, Beijing Forestry University, Beijing, China; ^4^China-USA Citrus Huanglongbing Joint Laboratory (GNU-UF Joint Lab), National Navel Orange Engineering Research Center, Gannan Normal University, Ganzhou, China; ^5^Linyi Inspection and Testing Center, Linyi, China

**Keywords:** citrus brown spot, virulence, conidiation, *Alternaria alternata*, histone methylation

## Abstract

Histone methylation, which is critical for transcriptional regulation and various biological processes in eukaryotes, is a reversible dynamic process regulated by histone methyltransferases (HMTs) and histone demethylases (HDMs). This study determined the function of 5 HMTs (*AaDot1*, *AaHMT1*, *AaHnrnp*, *AaSet1*, and *AaSet2*) and 1 HDMs (*AaGhd2*) in the phytopathogenic fungus *Alternaria alternata* by analyzing targeted gene deletion mutants. The vegetative growth, conidiation, and pathogenicity of ∆*AaSet1* and ∆*AaSet2* were severely inhibited indicating that *AaSet1* and *AaSet2* play critical roles in cell development in *A. alternata*. Multiple stresses analysis revealed that both *AaSet1* and *AaSet2* were involved in the adaptation to cell wall interference agents and osmotic stress. Meanwhile, ∆*AaSet1* and ∆*AaSet2* displayed serious vegetative growth defects in sole carbon source medium, indicating that *AaSet1* and *AaSet2* play an important role in carbon source utilization. In addition, ∆*AaSet2* colony displayed white in color, while the wild-type colony was dark brown, indicating *AaSet2* is an essential gene for melanin biosynthesis in *A. alternata*. *AaSet2* was required for the resistance to oxidative stress. On the other hand, all of ∆*AaDot1*, ∆*AaHMT1*, and ∆*AaGhd2* mutants displayed wild-type phenotype in vegetative growth, multi-stress resistance, pathogenicity, carbon source utilization, and melanin biosynthesis. To explore the regulatory mechanism of *AaSet1* and *AaSet2*, RNA-seq of these mutants and wild-type strain was performed. Phenotypes mentioned above correlated well with the differentially expressed genes in ∆*AaSet1* and ∆*AaSet2* according to the KEGG and GO enrichment results. Overall, our study provides genetic evidence that defines the central role of HMTs and HDMs in the pathological and biological functions of *A. alternata*.

## Introduction

Epigenetics is the study of heritable changes in gene expression that do not involve changes to the underlying DNA sequence, including histone variants, histone post-translational modifications, DNA methylation, and so on (Chinnusamy and Zhu, 2009). The basic subunit of eukaryotic chromatin is the nucleosome core particle, which consists of a core histone (containing two copies each of H3, H4, H2A, and H2B) octamer wrapped with 147 bp of DNA. Histone modification (such as methylation, acetylation, phosphorylation, and ubiquitination) of N-terminal tails mediated by specific enzymes is an important way of epigenetic regulation (Henikoff and Shilatifard, 2011). In the past 25 years, since the identification of the first histone acetyltransferase enzyme from *Tetrahymena thermophila* (Brownell and Allis, 1995; Brownell et al., 1996), the field of histone modification has grown quickly. As one of the most important ways of histone modification, histone methylation has been shown to play critical roles in fungal growth, cell development, multi-stress resistance, pathogenicity, and photoperiod regulation (Palmer et al., 2008; Lee et al., 2009; Connolly et al., 2013; Minh et al., 2015; Liu et al., 2016; Zhang et al., 2016; Gu et al., 2017a,b).

Histone methylation, which is mainly deposited on lysine (K) and arginine (R) residues of core histone N-terminal tails, is dynamically regulated by histone methyltransferases (HMTs) and histone demethylases (HDMs) and is essential in diverse biological processes ranging from transcriptional regulation to heterochromatin formation. At present, histone methylation analyses mainly focus on H3 and H4, in which K4, K9, K20, K27, K36, K79, R2, R3, R8, R17, and R26 are modified by mono/di/tri-methylation. Differences in the amino acid sites of histone methylation or the amount of added methyl group donors have been found to display various or even opposite effects on gene transcriptional regulation. For instance, H3K27me1 is commonly linked to transcriptional inhibition, while H3K27me3 tends to be associated with transcriptional activation (Laugesen et al., 2019). In addition, the function of transcriptional activation or inhibition mediated by H3K36me3 varies significantly by species (Strahl et al., 2002; Huang and Zhu, 2018). The number and amino acid sequence of HMTs and HDMs vary greatly in the genome of different species, even though histone proteins are highly conserved from yeast to animal. Lysine methylation of histone is carried out mainly by SET [Su(var)3–9, E(z) (enhancer of zeste) and trithorax] domain containing HMTs (Ng et al., 2002; Takahashi et al., 2011). The other class of HMTs, which does not contain SET domain, is represented by KMT4 (such as Dot1 in yeast; Van Leeuwen et al., 2002). Lysine demethylation of histone is catalyzed mainly by LSD domain containing HDMs (Shi et al., 2004).

At present, biological function studies on fungal histone methylation are mainly focused on several model fungi, such as *Saccharomyces cerevisiae*, *Neurospora crassa*, *Candida albicans*, and so on. In budding yeast *S. cerevisiae*, histone methylation has been shown to confer transcriptional memory of exposure to environmental stress conditions through mitotic divisions (Fabrizio et al., 2019). Loss of histone methylation at H3K4 triggers apoptosis in *S. cerevisiae* (Walter et al., 2014). In addition to gene transcriptional regulation, histone methylation also plays a critical role in regulating pre-RNA splicing in *S. cerevisiae* (Sorenson et al., 2016). Interestingly, histone methylation has been shown to be involved in DNA methylation in *N. crassa* (Tamaru and Selker, 2001; Honda et al., 2016). Methylation of H3K4 is required for *C. albicans* virulence by regulating intracellular level of reactive oxygen species (Kim et al., 2021). In filamentous pathogenic fungi, histone methylation has been found to be associated with pathogenicity, as well as vegetative growth, cell development, multi-stress resistance, and so on. In *Magnaporthe oryzae*, H3K4 methyltransferase *MoSET1* regulates global gene expression during infection-related morphogenesis (Minh et al., 2015). In *Fusarium graminearum*, *KMT6* histone methyltransferase regulates development and expression of secondary metabolite gene clusters (Connolly et al., 2013). In *Beauveria bassiana*, SET1/KMT2-governed H3K4 methylation coordinates the lifecycle of this fungal insect pathogen (Ren et al., 2021). In *Botrytis cinerea* and *Fusarium verticillioides*, H3K9 methyltransferase regulates pathogenicity, fungal development, and osmotic stress responses (Zhang et al., 2016; Gu et al., 2017a). In *Fusarium fujikuroi*, H3K36 methyltransferase Set2 is involved in regulating vegetative growth, sporogenesis, biosynthesis of secondary metabolites, and pathogenicity (Janevska et al., 2018).

*Alternaria alternata*, one of the most serious phytopathogenic fungi, harms citrus, apple, pear, strawberry, tobacco, tomato, and so on (Yang et al., 2017; Ma et al., 2018; Meng et al., 2018; Nishikawa and Nakashima, 2019; Song et al., 2019). Citrus brown spot (CBS), caused by the *A. alternata* tangerine pathotype, seriously harms citrus tender leaves, shoots, and fruits (Akimitsu et al., 2010). CBS often causes symptoms such as defoliation, shoot blight, and fruit-drop, which pose a serious threat to the citrus industry (Huang et al., 2015). Current research shows that deficiency of oxidative stress resistance, ACT-toxin biosynthesis, or vegetative growth will cause the virulence reduction of *A. alternata* (Gai et al., 2019, 2021; Ma et al., 2019, 2021; Chung et al., 2020). According to previous studies shown above, histone methylation is critical for the secondary metabolite biosynthesis, multi-stress resistance, vegetative growth, fungal development, and pathogenicity in other fungi. However, no functional analysis of histone methylation-related genes has been reported in *A. alternata*. Therefore, it is of great significance to conduct systematic identification of HMTs and HDMs, and biological function analysis of these genes in *A. alternata*.

In our previous study, we have demonstrated that *A. alternata* histone acetyltransferases and deacetylases are required for virulence, conidiation, DNA damage repair, and multi-stress resistance (Ma et al., 2021). In the present study, we identified all the HMTs and HDMs orthologs in *A. alternata* and clarified their roles in virulence, cell development, and multi-stress resistance. We also performed transcriptome analysis to explore the regulatory role of 2 HMTs (*AaSet1* and *AaSet2*) in this important citrus pathogen.

## Materials and Methods

### Fungal Strains and Culture Conditions

The wild-type Z7 strain of *A. alternata* (Fr.) Keissler used in this study was isolated from diseased citrus (*Citrus suavissima* Hort. Ex Tanaka) leaves in Zhejiang, China (Huang et al., 2015). All fungal strains were grown on PDA (potato dextrose agar) at 28°C for vegetative growth. Conidia were produced by culturing strains on V8 juice agar for 7–10 days. Fungal mycelium was collected from PDB (potato dextrose broth) by passing through cheesecloth and was used for RNA or DNA extraction. Fungal strains were cultured in Richard’s medium (Kohmoto et al., 1993) for 28 days for ACT toxin production.

### Gene Identification and Phylogenetic Analysis

Gene identification: Six HMTs (*AaDot1*, *AaHMT1*, *AaHnrnp*, *AaSet1*, *AaSet2*, and *AaSkb1*) and four HDMs (*AaGhd2*, *AaJhd1*, *AaRph1*, and *AaCyc8*) were identified combining the predicted results of hmm analysis and dbHiMo.^1^

Phylogenetic trees were constructed using the neighbor-joining algorithm of MEGA7 to compare amino acid sequences between different species.

### Targeted Gene Disruption

∆*AaDot1,* ∆*AaHMT1,* ∆*AaHnrnp,* ∆*AaSet1,* ∆*AaSet2,* and ∆*AaGhd2* strains were created by deleting *AaDot1, AaHMT1, AaHnrnp, AaSet1, AaSet2,* and *AaGhd2*, respectively, by integrating a bacterial *HYG* cassette under control of *TrpC* gene promoter and terminator in the genome of Z7 using a split marker approach mediated by protoplast transformation as described previously (Li et al., 2021; Gai et al., 2022). Fungal transformants were recovered from PDA containing 100 μg/ml hygromycin and were checked by PCR with primers specific to its targeted gene. Oligonucleotide primers used in this study are listed in Supplementary Table 1.

### Sensitivity Tests

Chemical sensitivity analysis was conducted by transferring fungal mycelia by sterile toothpicks to PDA amended with tested compound and incubated at 28°C for 3–6 days. Tested compounds included H_2_O_2_ (10 mm), sorbitol (1 M), KCl (1 M), NaCl (1 M), CaCl_2_ (250 mm), CuSO_4_ (1 mm), sodium dodecyl sulfate (SDS, 0.01%), Congo red (CR, 0.2 mg/ml), chlorothalonil (10 mg/l), Mancozeb (2 mg/l), Boscalid (3 mg/l), hydroxyurea (HU, 5 mm), or Camptothecin (CPT, 5 μm). To examine the carbon utilization ability, mutants and wild type were inoculated on MM medium (Gai et al., 2021) using glucose (20 g/l), sucrose (10 g/l), starch (10 g/l), or lactose (10 g/l) as sole carbon source. All tests were repeated at least twice with three replicates of each treatment. Ratio change was calculated by dividing the relative difference of the growth with and without stress by the growth without the corresponding stress. All test chemicals were purchased from Sigma-Aldrich (St. Louis, MO, United States).

### Virulence Assays

Fungal virulence was assessed on detached Dancy (*Citrus reticulata* Blanco) leaves inoculated by placing a 5-mm dia. Agar plug covered with fungal mycelium on each spot, and those inoculated leaves were kept in a plastic box at 28°C for 2–4 days for lesion development.

The method for ACT toxin virulence analysis was used same with previous study (Ma et al., 2019).

### Gene Expression Analyses

Quantitative real-time PCR (qRT-PCR) was carried out on a Q2000A Real Time PCR system (Hangzhou Langji Scientific Instrument Co., LTD) to measure gene expression levels. RNA was extracted with Trizol (R401-01, Vazyme). cDNA was synthesized from RNA using HiScript III 1st Strand cDNA Synthesis Kit (+gDNA wiper; R312-01, Vazyme). qRT-PCR was performed using ChamQ SYBR qPCR Master Mix (Q321-02, Vazyme). A comparative ∆Cτ method was used to calculate relative expression levels (Schefe et al., 2006). Each treatment was repeated at least twice with three replicates.

### Transcriptome Sequencing and Analysis

Wild type, ∆*AaSet1,* and ∆*AaSet2* each with six replicates were cultured in PDB at 28°C in dark condition at 120 rpm for 36 h. Three replicates of each strain were harvested by passing through cheesecloth. For the other three replicates of each strain, mycelia were treated with detached Dancy leaves for 30 min and were harvested by passing through cheesecloth. RNA extraction of these collected mycelium was conducted using Trizol method mentioned above. RNA-seq libraries were generated using an NEBNext Ultra RNA library prep kit for Illumina (catalog no. E7530L; NEB, United States). Sequencing was conducted using Illumina HiSeq platform, and 150-bp paired-end reads were generated. The *A. alternata* Z7 genome (Wang et al., 2016) was used as the reference genome. Adaptors and low-quality bases of raw data were removed using Trimmomatic v0.36 (Bolger et al., 2014). HISAT2 v2.1.0 was used to map clean reads to the reference genome (Kim et al., 2015). Count matrix of genes was generated using Htseq-count (Anders et al., 2015). Differentially expressed genes (DEGs) were generated using DESeq2 under threshold of FDR < 0.05 and |log_2_^FC^| ≥ 1 (Love et al., 2014). GO and KEGG pathway enrichment were conducted using WEGO (Web Gene Ontology Annotation Plot 2.0; Ye et al., 2006) and KEGG (Kyoto Encyclopedia of Genes and Genomes; Kanehisa and Goto, 2000), respectively.

### Data Availability

All the RNA-seq data have been deposited at NCBI under the BioProject accession number (PRJNA813997). All the amino acid sequences of HMTs and HDMs in fungi used in this study can be found in Supplementary Tables 2, 3.

### Data Analysis Methods

Unless otherwise indicated, all experiments were conducted at least two times with three replicates. The statistical significance of treatments was determined by analysis of variance and means separated by Duncan’s test (*p* < 0.05).

## Result

### Identification and Gene Disruption of HMTs and HDMs in *A. alternata*

**Figure 1:**
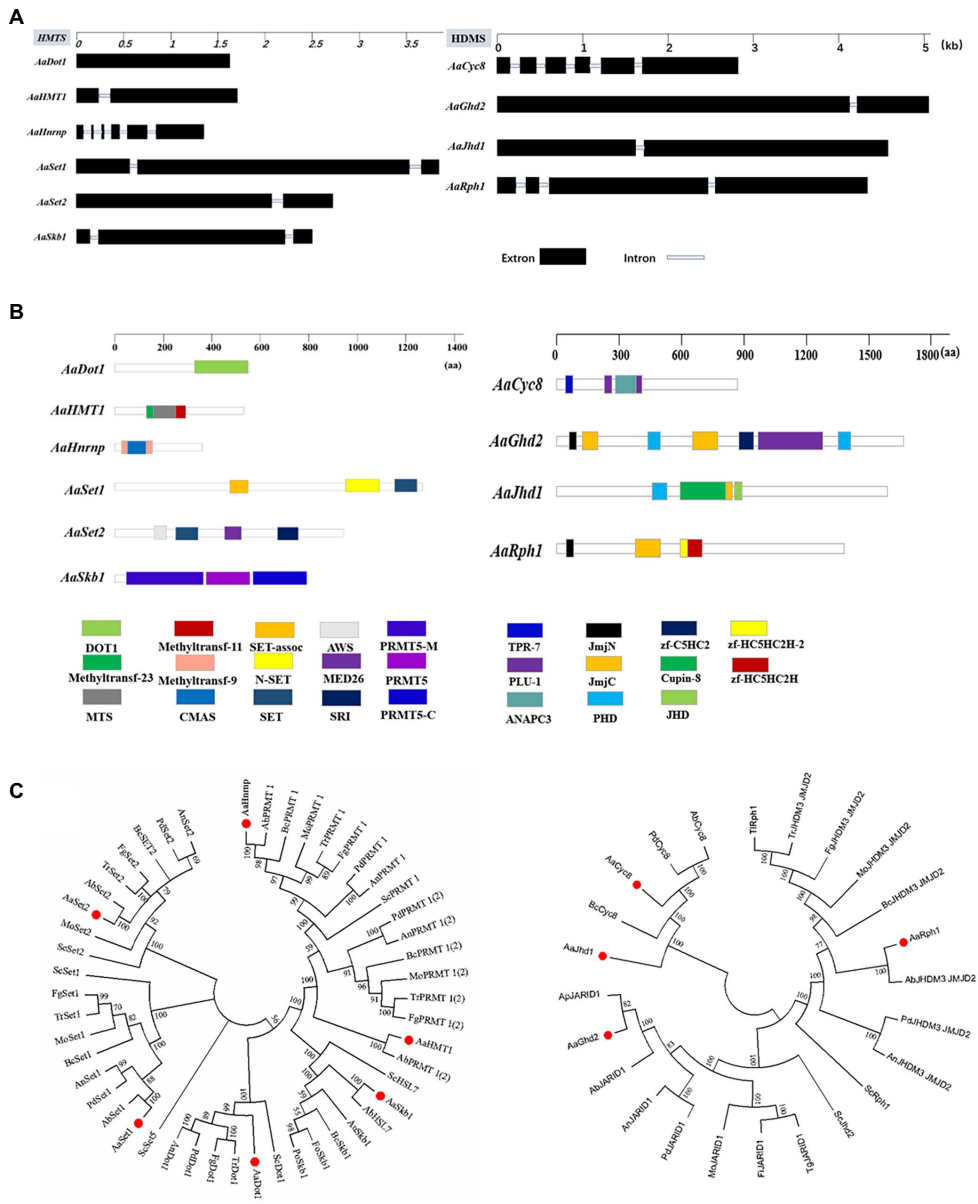
Identification and phylogenetic analysis of HMTs and HDMs in *A. alternata.* **(A)** Schematic depiction of the gene structure of HMTs and HDMs. **(B)** Schematic depiction of conserved protein domains of HMTs and HDMs. **(C)** Phylogenetic trees were constructed using the neighbor-joining algorithm of MEGA7. (Aa: *A. alternata*; Ab (left): *A. brassicicola*; Bc: *B. cinerea*; Mo: *M. oryzae*; Tr: *T. reesei*; Fg: *F. graminearum*; Pd: *P. digitatum*; An: *A. nidulans*; Fo: *F. oxysporum*; Au: *A. udagawae*; Ap: *A. panax*; Tg: *T. gamsii*; Fl: *F. longipes*; Tl: *T. lentiforme*; Ab (right): *A. bombycis*).

Six HMTs (*AaDot1*, *AaHMT1*, *AaHnrnp*, *AaSet1*, *AaSet2*, and *AaSkb1*) and four HDMs (*AaGhd2*, *AaJhd1*, *AaRph1*, and *AaCyc8*) listed in Table 1 were identified combining the predicted results of hmm analysis and dbHiMo. Nucleotide sequence length of corresponding predicted genes ranges from 1608 to 5013 bp, the number of introns of these genes varies from 0 to 5, and the length of corresponding proteins varies from 507 to 1654 aa (Figures 1A,B). Functional domain prediction of amino acid sequences revealed that all these 10 proteins contain functional domains unique to HMT or HDM, indicating the prediction of these genes is highly reliable. Chromosome location analysis showed that all predicted HMTs and HDMs are localized in essential chromosomes, whereas no genes were identified in CDC (conditionally dispensable chromosomes; Supplementary Figure 1). Phylogenetic analysis revealed that all predicted HMTs and HDMs of *A. alternata* were highly similar to the homologous genes in model or other filamentous fungi (Figure 1C).

**Table 1:** HMTs and HDMs in *Alternaria alternata.*

Gene	Accession no	Length (nt)	Type	Deletion mutants	Number of mutants	Vegetative growth
*AaDot1*	AAL_g3463	1,608	HMTs	*AaDot1*	21	Normal
*AaHMT1*	AAL_g2753	1,597	HMTs	*AaHMT1*	18	Normal
*AaHnrnp*	AAL_g5237	1,322	HMTs	*AaHnrnp*	17	Slightly inhibited
*AaSet1*	AAL_g7533	3,944	HMTs	*AaSet1*	48	Inhibited
*AaSet2*	AAL_g4603	2,838	HMTs	*AaSet2*	45	Inhibited
*AaSkb1*	AAL_g4722	2,500	HMTs	*AaSkb1*	0	No information
*AaGhd2*	AAL_g2163	5,013	HMTs	*AaGhd2*	18	Normal
*AaJhd1*	AAL_g9119	4,751	HMTs	*AaJhd1*	0	No information
*AaRph1*	AAL_g4564	4,315	HMTs	*AaRph1*	0	No information
*AaCyc8*	AAL_g9253	2,840	HMTs	*AaCyc8*	0	No information

To investigate the function of HMTs and HDMs, mutants defective for *AaDot1* (∆*AaDot1*), *AaHMT1* (∆*AaHMT1*), *AaHnrnp* (∆*AaHnrnp*), *AaSet1* (∆*AaSet1*), *AaSet2* (∆*AaSet2*) and *AaGhd2* (∆*AaGhd2*) were generated using split marker system (Supplementary Figure 2A). All the corresponding mutants were verified by PCR analysis (Supplementary Figure 2B). However, no gene deletion mutants of *AaSkb1*, *AaJhd1*, *AaRph1,* and *AaCyc8* were obtained after several attempts.

### HMTs Are Required for Vegetative Growth and Fungal Development

**Figure 2:**
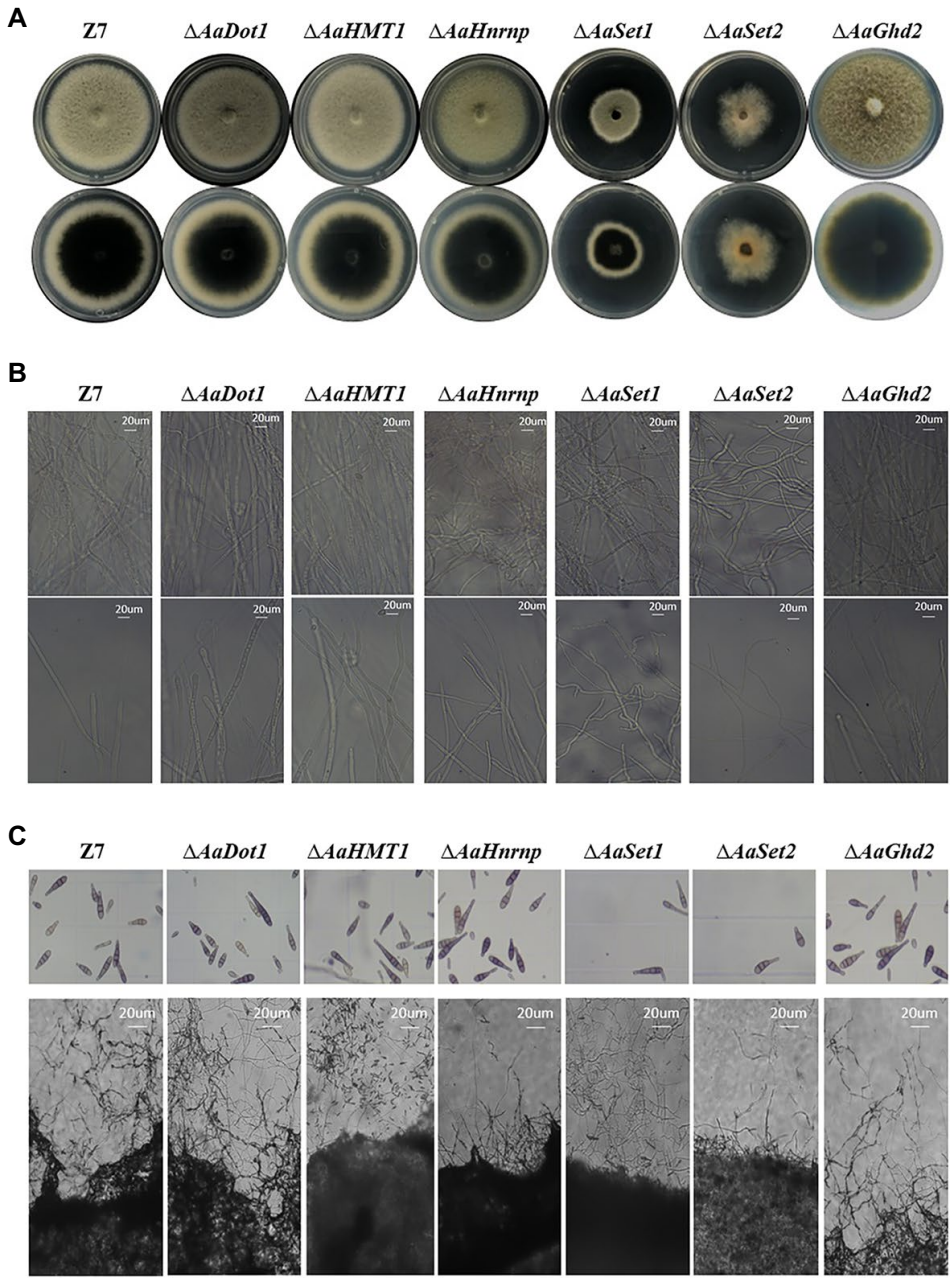
HMTs were required for vegetative growth and fungal development. **(A)** Radial growth of mutants and wild-type strains 4 days post-incubation. Z7: the wild-type strain. Upper panel shows the back side of the corresponding colonies, and the lower panel shows the front side of the colonies. **(B)** Hyphae morphology of mutants and wild-type strains. Upper panel represents areas with dense mycelium distribution, and the lower panel represents the mycelium morphology at the edge of corresponding colonies. **(C)** Conidiation of mutants and wild-type strain. Upper panel represents the eluted spores, and the lower panel represents the spores before elution.

In disrupted mutants of HMTs and HDMs, vegetative growth rate of ∆*AaSet1* and ∆*AaSet2* was reduced by 43 and 36% compared to the wild-type strain on PDA, indicating that *AaSet1* and *AaSet2* play a critical role on vegetative growth (Figure 2A; Supplementary Figure 3). Fewer aerial hyphae of ∆*AaSet2* were produced on PDA compared to wild-type strain. In addition, with the prolongation of time, most of the mutant colonies turned into dark brown color, while the colony of ∆*AaSet2* was still white, indicating *AaSet2* was an essential gene for melanin biosynthesis of *A. alternata* (Figure 2A). In contrast to ∆*AaSet2*, the colony of ∆*AaSet1* was darker than wild type, indicating *AaSet1* negatively regulates the melanin biosynthesis. Microscopic examination revealed that the impacts of different HMTs on the development of *A. alternata* varied greatly. The ∆*AaSet1* and ∆*AaSet2* strains produced severely curved hyphae (Figure 2B), and the sporulation ability was seriously inhibited (Figure 2C; Supplementary Figure 4). The hyphae of the ∆*AaHnrnp* strain was slightly curved, but its sporulation ability was not affected. The ∆*AaDot1*, ∆*AaHMT1,* and ∆*AaGhd2* displayed wild-type levels of vegetative growth and cell development.

### HMTs and HDMs Are Involved in Resistance to Multiple Stresses

**Figure 3:**
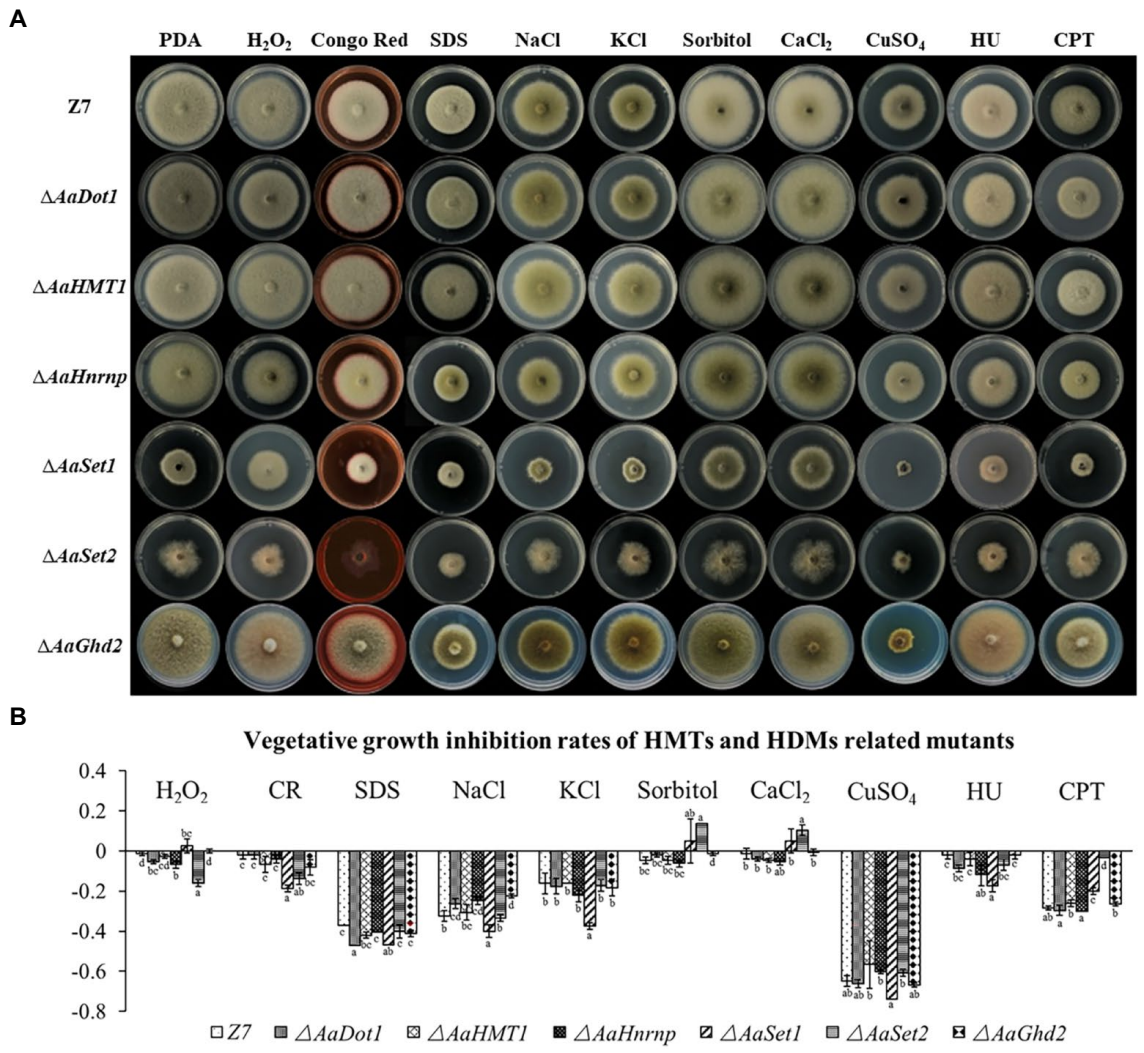
Chemical sensitivity assays. **(A)** HMTs- and HDMs-related genes knock out mutants and wild-type Z7 was grown on PDA amended with or without the indicated chemicals. **(B)** Quantitative analysis (the growth inhibition rate of strains on PDA amended with tested chemicals compared to corresponding strains on PDA) of mutants and wild type. Computational formula: [dia._(Z7 or mutant under stress)_−dia. _(Z7 or mutant in PDA)_]/dia. _(Z7 or mutant in PDA)_.

Vegetative growth of ∆*AaDot1*, ∆*AaHnrnp,* and ∆*AaSet2* strain was significantly inhibited in PDA containing 10 mm H_2_O_2_, and the growth inhibition rate was increased by 5, 7 and 16% compared to wild type, respectively (Figure 3). However, ∆*AaHMT1* and ∆*AaGhd2* had no significant difference in vegetative growth inhibition rate compared with wild type on the medium containing H_2_O_2_. These results indicated that *AaDot1*, *AaHnrnp,* and *AaSet2* were important genes in the oxidative stress resistance pathway of *A. alternata*. In contrast, ∆*AaSet1* increased radial growth when PDA was amended with 10 mm H_2_O_2_ (Figure 3), indicating *AaSet1* may negatively regulate the oxidative stress resistance. ∆*AaSet1* displayed 47% growth reduction on PDA amended with SDS (sodium dodecyl sulfonate, cell wall-interfering agent). In addition, the growth inhibition rate of both ∆*AaSet1* and ∆*AaSet2* on PDA amended with CR (Congo red, cell wall-interfering agent) increased significantly, indicating both *AaSet1* and *AaSet2* is involved in cell wall integrity maintenance (Figure 3). The sensitivity of ∆*AaSet1* and wild type to 1 M sorbitol was similar. Interestingly, when PDA was amended with 1 M NaCl or 1 M KCl, ∆*AaSet1* reduced radial growth by nearly 40 and 38%, while the wild type just reduced by 32% (1 M NaCl) and 16% (1 M KCl), respectively, indicating *AaSet1* plays critical roles in the resistance of salt stresses rather than osmotic stress (Figure 3). In contrast to ∆*AaSet1*, ∆*AaSet2* displayed highly enhanced resistance to sorbitol but similar sensitivity to salt stresses compared to wild type, indicating *AaSet2* negatively regulates the resistance to osmotic stress but is not involved in the resistance to salt stresses. However, the addition of CuSO_4_ (1 mm) into PDA caused similar growth inhibition rate of all the mutants and wild type, indicating *AaDot1*, *AaHMT1*, *AaHnrnp*, *AaSet1*, *AaSet2*, and *AaGhd2* are not involved in the adaption of CuSO_4_. ∆*AaSet2* increased radial growth on PDA amended with CaCl_2_ (250 mm), while wild type as well as the other mutants displayed reduced radial growth under same condition, indicating *AaSet2* negatively regulates the adaption to CaCl_2_. In the assay of DNA damage-inducing agent sensitivity, the growth inhibition rate of ∆*AaSet1* on hydroxyurea (HU) containing medium was significantly higher than that of wild type. On the other hand, the growth inhibition rate of ∆*AaSet1* and ∆*AaSet2* on camptothecin (CPT) containing medium was lower (−20% and − 3%) than that of wild type (−28%), indicating *AaSet1* and *AaSet2* are involved in the adaption of DNA damage-inducing agents (Figure 3).

### HMTs Are Required for the Utilization of Carbon Sources and Fungicide Resistance

**Figure 4:**
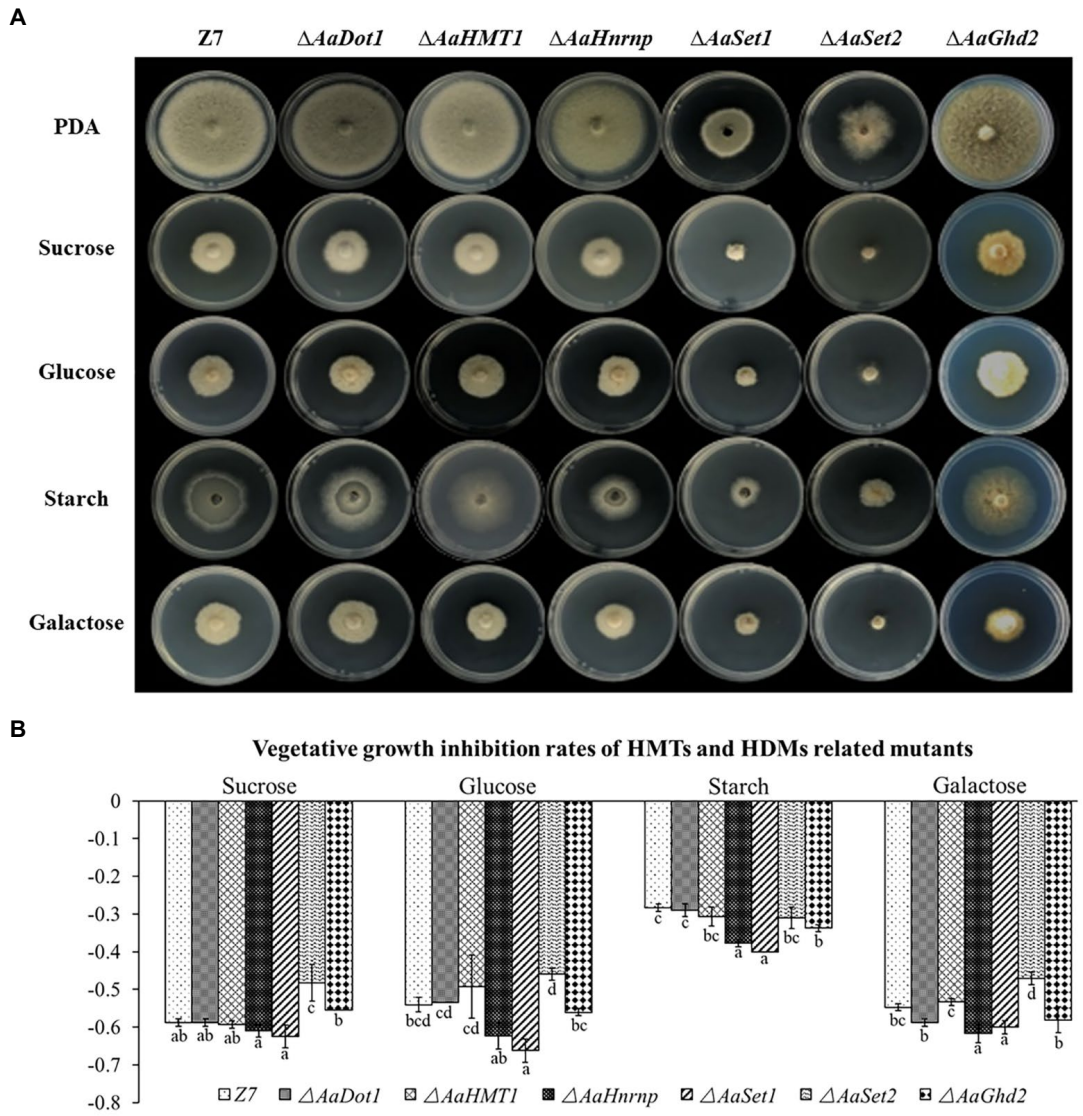
**(A)** HMTs were involved in carbon source utilization. Z7 and gene deletion mutants cultured on MM medium amended with sucrose, glucose, galactose, or starch as sole carbon source. **(B)** Growth inhibition rate of strains on corresponding medium. Computational formula: [dia._(Z7 or mutant under stress)_−dia. _(Z7 or mutant in PDA)_]/dia. _(Z7 or mutant in PDA)_.

To examine whether HMTs or HDMs were required for the utilization of carbon sources, mycelial radical growth assays were performed on MM medium supplemented with sucrose (10 g/l), starch (10 g/l), glucose (20 g/l), or galactose (10 g/l) as sole carbon sources (Figure 4). Compared with the wild type, the radial growth of both ∆*AaHnrnp* and ∆*AaSet1* was severely inhibited when the MM medium amended with glucose, starch, or galactose as sole carbon source, indicating *AaHnrnp* and *AaSet1* are required for the utilization of glucose, starch, or galactose. In contrast, *AaSet2* negatively regulates the utilization of sucrose and galactose. Other gene disruption mutants, including ∆*AaDot1*, ∆*AaHMT1,* and ∆*AaGhd2*, exhibited wild-type sensitivity to all test medium, indicating that these genes are not involved in the utilization of sucrose, glucose, starch, and galactose.

**Figure 5:**
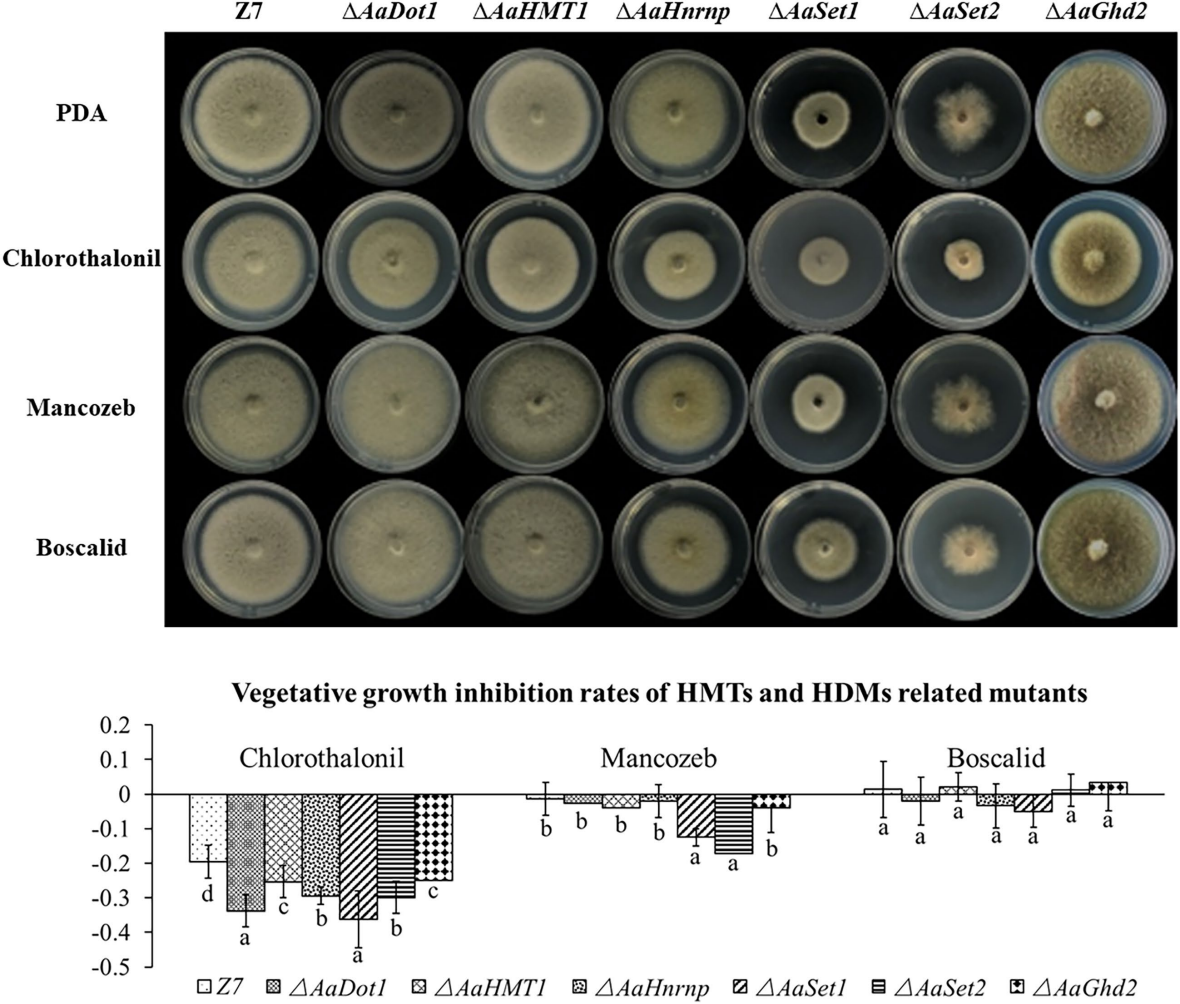
HMTs were involved in fungicide resistance. Computational formula: [dia._(Z7 or mutant under stress)_−dia. _(Z7 or mutant in PDA)_]/dia. _(Z7 or mutant in PDA)_.

The vegetative growth of ∆*AaSet1* and ∆*AaSet2* on medium containing mancozeb was more seriously inhibited (−12.5% and − 17.2%) than that of the wild type (−1.3%), indicating that both of these genes are involved in mancozeb resistance (Figure 5).

### HMTs and HDMs Are Required for Fungal Virulence

**Figure 6:**
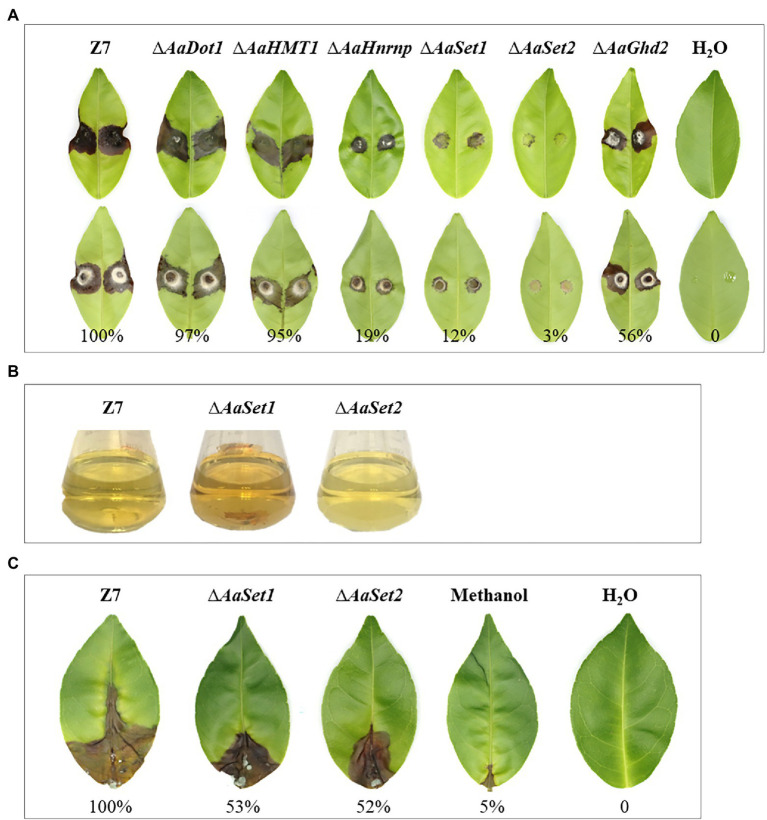
HMTs and HDMs are required for fungal virulence. **(A)** Virulence assays of strains. Upper panel represents the front side of inoculated leaves, and the back side of inoculated leaves. **(B)** Filtrates of ∆*AaSet1*, ∆*AaSet2* and wild type. **(C)** ACT toxin virulence analysis of ∆*AaSet1*, ∆*AaSet2* and wild type.

Fungal virulence analyses on detached Dancy leaves were evaluated using wild type and all mutants generated in this study. Virulence assays revealed a reduction of necrotic lesions induced by ∆*AaHnrnp*, ∆*AaSet1,* and ∆*AaGhd2* mutants compared to those induced by wild-type 2 days post-inoculation (dpi). In addition, ∆*AaSet2* almost failed to induce visible lesion on detached Dancy leaves, indicating *AaSet2* is essential for fungal infection (Figure 6A). The pathogenicity capability of ∆*AaDot1* and ∆*AaHMT1* is similar to that of wild type on Dancy leaves 2 dpi. Quantitative analysis revealed that the necrotic lesion area induced by ∆*AaDot1*, ∆*AaHMT1*, ∆*AaHnrnp*, ∆*AaSet1*, ∆*AaSet2*, and ∆*AaGhd2* was about 97, 95, 19, 12, 3, and 56%, respectively, of those induced by the wild type.

In view of the deficiency in vegetative growth, sporulation, and virulence of ∆*AaSet1* and ∆*AaSet2*, we performed ACT toxin extraction of ∆*AaSet1*, ∆*AaSet2,* and wild type. The filtrate color of ∆*AaSet1*, ∆*AaSet2,* and wild type varied greatly. The filtrate of ∆*AaSet2* displayed a clear color and ∆*AaSet1* displayed a darker color, as compared to those induced by wild type (Figure 6B). ACT toxin virulence of ∆*AaSet1* and ∆*AaSet2* decreased significantly, suggesting that *AaSet1* and *AaSet2* were involved in ACT toxin biosynthesis process of *A. alternata* (Figure 6C).

### Transcriptome Analysis Defines the Global Regulatory Role of *AaSet1* and *AaSet2*

The results mentioned above revealed that both *AaSet1* and *AaSet2* are involved in vegetative growth, fungal infection, cell development, carbon sources utilization, multi-stress resistance, ROS detoxification, and melanin biosynthesis. Therefore, transcriptomic analysis of ∆*AaSet1* and ∆*AaSet2* compared to wild type was conducted to explore the transcriptional regulatory mechanism of *AaSet1* and *AaSet2*. Wild type, ∆*AaSet1,* and ∆*AaSet2* each with three replicates were incubated in PDB, or PDB containing detached citrus leaves. Illumina libraries were constructed from wild type, ∆*AaSet1,* and ∆*AaSet2* with or without the induction of citrus leaves and sequenced using HiSeq platform. The number of clean reads in each sample was more than 44,000,000, accounting for more than 94% of raw reads (Supplementary Table 4). Under PDB condition, 2,615 differentially expressed genes (DEGs) consisting of 1,700 upregulated and 915 downregulated genes were identified in ∆*AaSet1* compared to wild type (Supplementary Tables 5, 6). Under citrus leaves induction treatment, 1873 upregulated and 881 downregulated genes were identified in ∆*AaSet1* compared with wild type (Supplementary Tables 5, 7). Under PDB condition, 1880 upregulated and 2,600 downregulated genes were identified in ∆*AaSet2* compared to wild type (Supplementary Tables 5, 8). Under citrus leaves induction treatment, 1917 upregulated and 2,727 downregulated genes were identified in ∆*AaSet2* compared to wild type (Supplementary Tables 5, 9).

**Figure 7:**
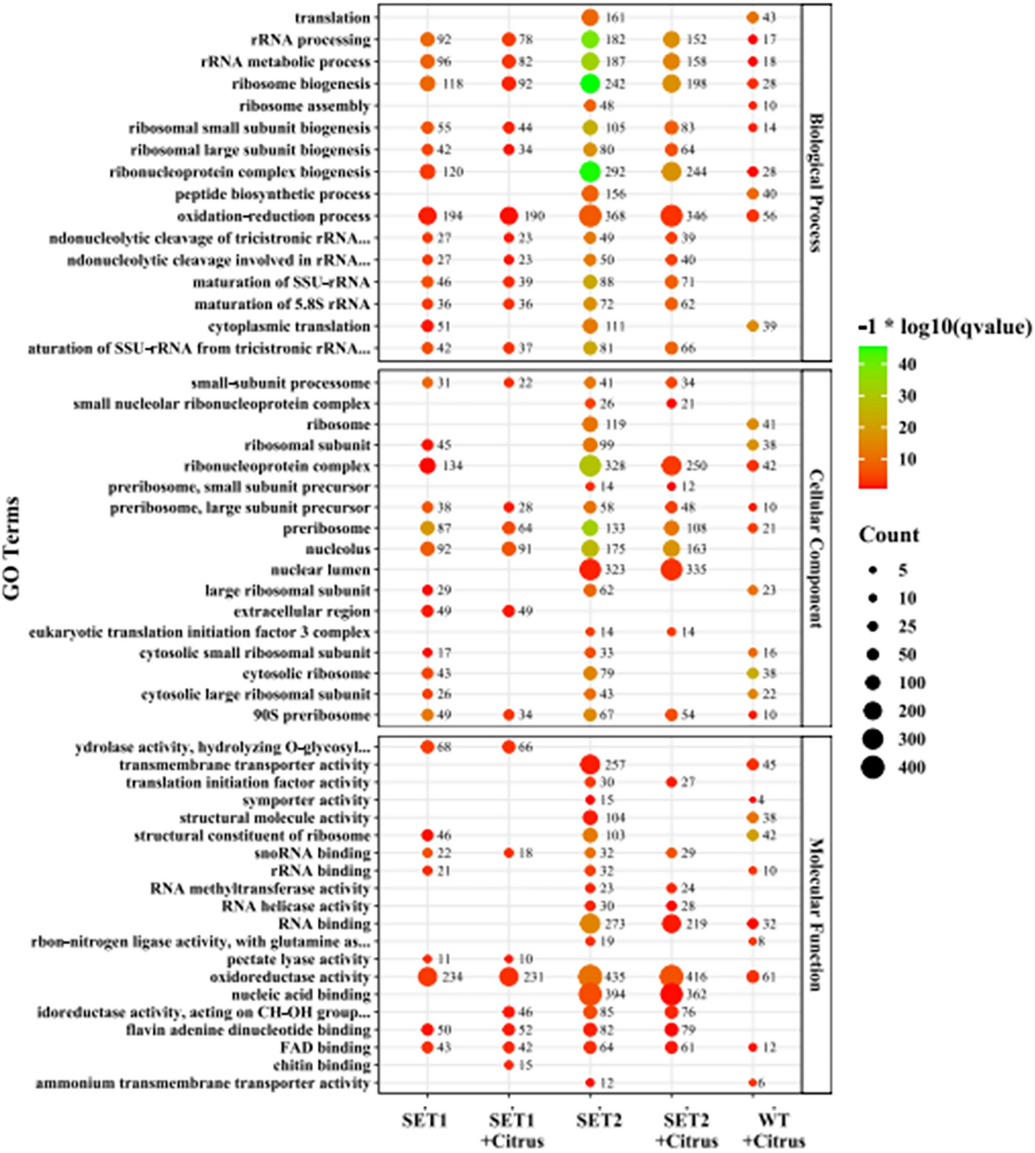
GO enrichment analysis of Δ*AaSet1* and Δ*AaSet2.*

GO (Gene Ontology) analysis of ∆*AaSet1* and ∆*AaSet2* revealed that many DEGs were enriched in the metabolism category, such as Ribosomal RNA processing, ribosomal biosynthesis, ribosomal assembly, translation, ribosomal assembly, and peptide biosynthesis processes. There are also many DEGs in ∆*AaSet1* and ∆*AaSet2* enriched in hydrolase activity, redox enzyme activity, transmembrane transporter activity, cotransporter activity, structural molecule activity, RNA binding, and nucleic acid binding function (Figure 7; Supplementary Tables 10, 11).

**Figure 8:**
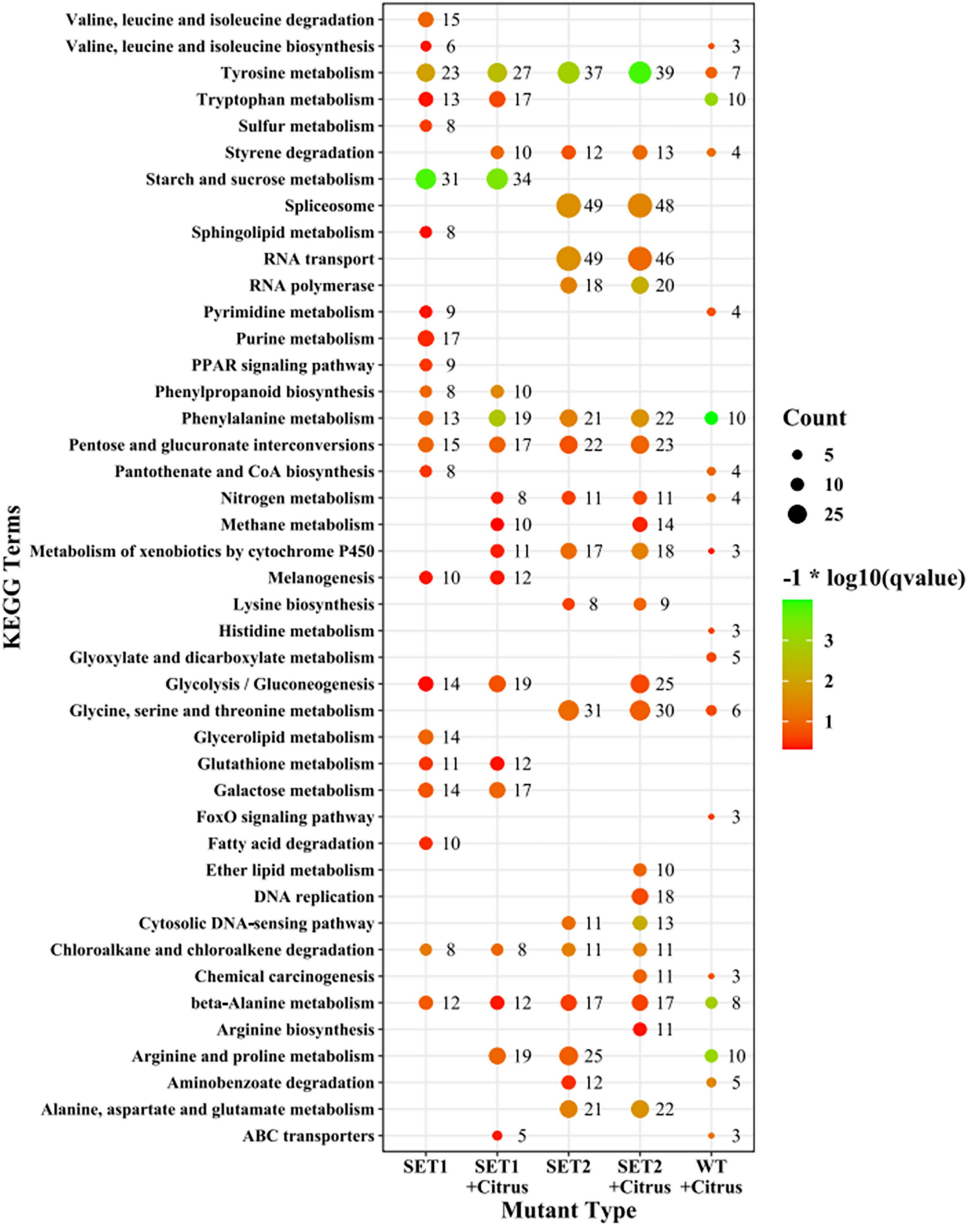
KEGG enrichment analysis of Δ*AaSet1* and Δ*AaSet2.*

KEGG enrichment analysis revealed that the most affected metabolism-related pathways in ∆*AaSet1* were mainly related to the metabolism pathways of valine, leucine and isoleucine, pyrimidine and purine, fatty acid degradation, phenylpropane synthesis, glutathione metabolism, galactose metabolism, and so on. The most affected metabolism-related pathways in ∆*AaSet1* were mainly involved in RNA transport, glycine, serine, and threonine metabolism (Figure 8; Supplementary Tables 12, 13).

### *AaSet1* and *AaSet2* Are Required for Transcriptional Regulation of Genes Involved in RNA Processing and Translation

A large number of upregulated DEGs in ∆*AaSet2* are enriched in RNA polymerase (ko03020), spliceosome (ko03040), ribosome biosynthesis (ko03010), and lysine biosynthesis (ko00300) related pathways (Supplementary Figures 5–8). ko03020 is involved in RNA biosynthesis; ko03040 is involved in RNA splicing; ko03010 is required for translating RNA into protein; the lysine synthesis in ko00300 is important for histone methylation, indicating *AaSet2* plays an important role in the gene transcription, RNA processing and translation. In addition, *AaSet2* negatively regulates the synthesis of its catalytic site (lysine). In contrast to ∆*AaSet2*, DEGs of ∆*AaSet1* are mainly enriched in ribosome-related pathways (ko03010 and ko03008; Supplementary Figures 9, 10).

### *AaSet1* and *AaSet2* Regulate Transcription of ROS Detoxification Genes

ROS homeostasis is critical for the regulation of multiple biological processes in cells. In this study, we analyzed the expression of 96 antioxidation genes in ∆*AaSet1* and ∆*AaSet2*, including 20 genes in thioredoxin system, 42 genes in glutathione system, 7 genes encoding superoxide dismutase (SOD), 7 genes encoding catalases, and 20 peroxidases (Supplementary Table 14). In ∆*AaSet1*, 11 DEGs were downregulated and 21 were upregulated when cultured in PDB, and 9 DEGs were downregulated and 19 were upregulated when induced by citrus leaves. Of these ROS detoxification-related DEGs, the number of upregulated DEGs was approximately twice that of downregulated DEGs, and all DEGs of catalases and peroxidase were upregulated. In ∆*AaSet2*, 29 DEGs were downregulated and 13 were upregulated when cultured in PDB, and 32 DEGs were downregulated and 11 were upregulated when induced by citrus leaves. Of these ROS detoxification-related DEGs, the number of downregulated DEGs was approximately three times that of upregulated DEGs and all DEGs of catalases were downregulated. Combined with the experimental results, the H_2_O_2_ resistance capability of ∆*AaSet1* was increased, while ∆*AaSet2* was decreased, indicating that both *AaSet1* and *AaSet2* maintained *A. alternata* ROS homeostasis *via* regulating the transcription of ROS detoxification genes.

### *AaSet1* and *AaSet2* Regulate Expression of Genes Involved in CAZymes, CYP450, Secondary Metabolite Cluster, and Conidiation

**Figure 9:**
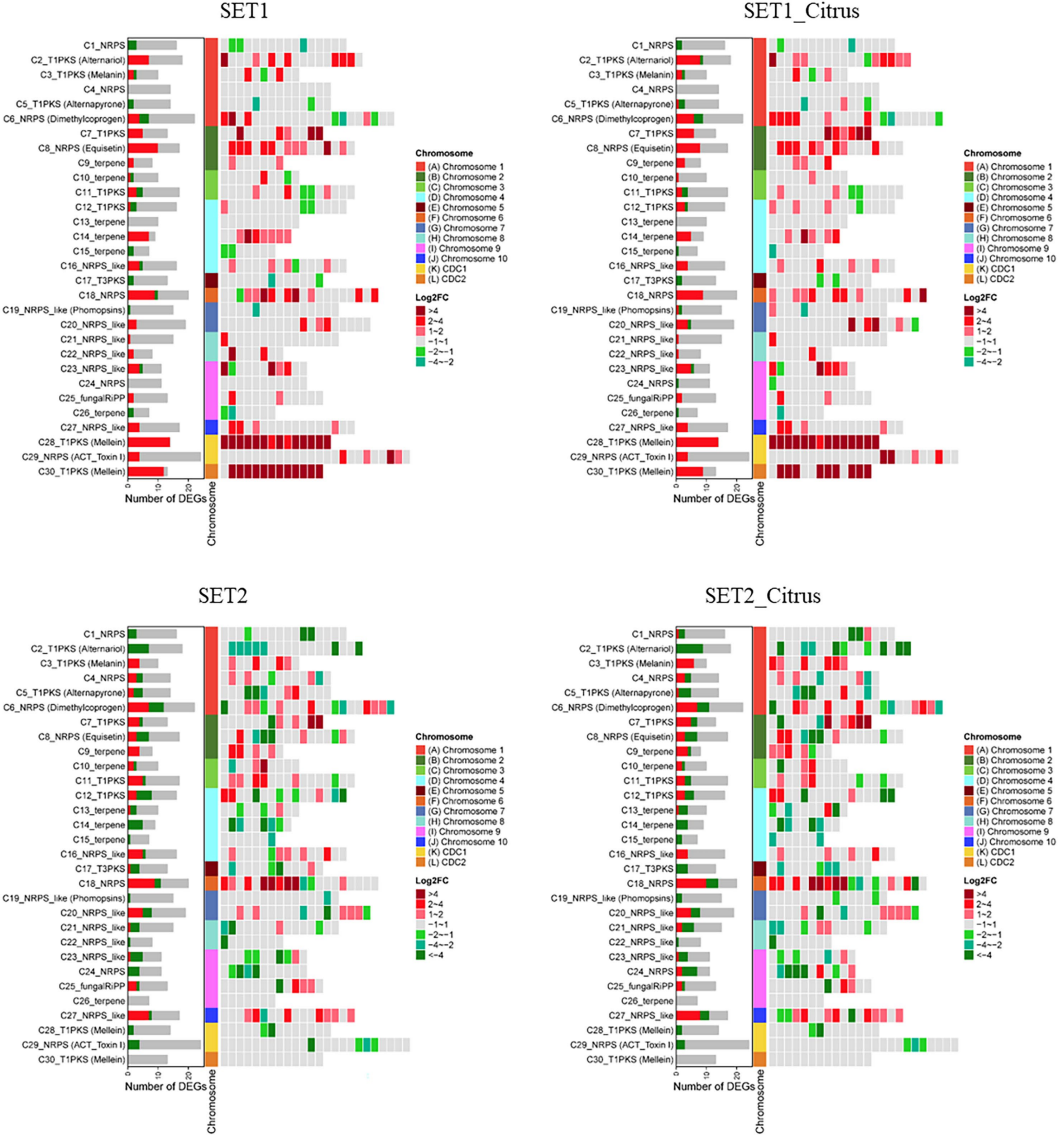
Differential expression of gene clusters associated with the biosynthesis of secondary metabolites in ∆*AaSet1* and ∆*AaSet2* compared to wild type with or without the induction of citrus leaves.

Secondary metabolite biosynthesis gene clusters predicted using antiSMASH 4.0 revealed that *AaSte1* and *AaSte2* regulates numerous genes in these gene clusters (Figure 9; Supplementary Figures 11–14). As one of the most important secondary metabolites, ACT is essential for the virulence of *A. alternata*. In ∆*AaSet1*, all DEGs in ACT toxin gene cluster were upregulated, including ACTTS3 (AALT_g11750), ACTT5 (AALT_g11751), ACTTR (AALT_g11754), polyketide synthase (AALT_g11757), and cytochrome P450 monooxygenase (AALT_g12047). In contrast to ∆*AaSet1*, all DEGs of ∆*AaSet2* in ACT toxin gene cluster were downregulated, including ACTTR, ACTT3, and two hypothetical protein encoding genes (AALT_g11747 and AALT_g11753; Supplementary Table 15). Cell wall-degrading enzyme is also critical for the virulence of *A. alternata* (Ma et al., 2019). Deletion of *AaSet1* or *AaSet2* significantly affected the expression of genes related to carbohydrate-active enzymes (CAZymes), and the total number of DEGs exceeded 200 and 300, respectively (Supplementary Table 16). In ∆*AaSet2*, which is incubated in PDB, all seven DEGs of cutinase (AALT_g943, AALT_g9295, AALT_g9279, AALT_g5432, AALT_g4955, AALT_g4174, and AALT_g1915) were downregulated. Some genes encoding P450 or involved in conidiation were also differentially expressed in ∆*AaSet1* and ∆*AaSet2* (Supplementary Tables 17, 18).

## Discussion

In eukaryotic organisms, the readout of genetic information is significantly regulated by modifications of histone. Histone methylation was first revealed more than half a century ago (Allfrey et al., 1964). In recent years, research on histone methylation is increasing rapidly, and it has been found that such modification plays critical roles in multiple biological processes by regulating gene transcription (Bannister and Kouzarides, 2005). Histone methylation level manifested by HMTs and HDMs dynamically changes for transcriptional regulation of specific genes. Previous studies revealed that HMTs and HDMs are required for vegetative growth, cell development, multi-stress resistance, secondary metabolite biosynthesis, or pathogenicity in fungi (Connolly et al., 2013; Minh et al., 2015; Zhang et al., 2016; Gu et al., 2017a; Kim et al., 2021; Ren et al., 2021). However, studies of histone methylation are still rarely reported in phytopathogenic fungi, although it has been extensively analyzed in model species. In addition, no report of systematic identification and gene function analysis of HMTs or HDMs family genes in a fungus can be found. In this study, we identified a total of 6 HMTs and 4 HDMs in *A. alternata*. Subsequently, we knocked out five HMTs (*AaDot1*, *AaHMT1*, *AaHnrnp*, *AaSet1*, and *AaSet2*) and one HDMs (*AaGhd2*) and obtained more than three independent transformants of each gene. Gene function analysis using these transformants revealed that HMTs are extensively involved in transcriptional regulation, hyphal growth, conidiation, multi-stress resistance, melanin biosynthesis, and virulence in *A. alternata*.

Our previous studies revealed that the capability of ROS detoxification (Ma et al., 2018, 2021; Gai et al., 2019), ACT toxin biosynthesis (Li et al., 2021), and cell wall-degrading enzymes (Ma et al., 2019) are required for the full virulence of *A. alternata*. In this study, the virulence of ∆*AaHnrnp*, ∆*AaSet1*, ∆*AaSet2,* and ∆*AaGhd2* decreased significantly. Although the vegetative growth rate of ∆*AaSet1* and ∆*AaSet2* was similar in PDA medium, ∆*AaSet2* was less virulent than ∆*AaSet1*, indicating that *AaSet1* and *AaSet2* regulate pathogenicity *via* different mechanism. The ROS detoxification capability of ∆*AaSet2* was significantly decreased, indicating that *AaSet2* positively regulates ROS detoxification. In addition, numerous genes involved in ROS detoxification (encoding for catalase, SOD, thioredoxin, glutathione, and peroxidase and so on), ACT toxin biosynthesis, and fungal penetration (cutinase, cellulase, and pectate lyase) were significantly downregulated, indicating *AaSet2* may regulate the pathogenicity of *A. alternata* through transcriptional regulation of genes in multiple pathways. In contrast to ∆*AaSet2*, ∆*AaSet1* is slightly resistant to H_2_O_2_. The histone methylation network is extensively conserved among eukaryotes, from the methylation sites to enzymatic machinery responsible for their regulation (Roguev et al., 2003; Fuchs et al., 2006; Woo and Li, 2012). The functioning of HMTs and HDMs depends on specific domain. HMTs mainly contains SET or Dot1 domain. In the two best-studied fungi of *S. pombe* (Sp) and *S. cerevisiae* (Sc), SpSet1, SpSet2, SpSet4, SpSet9, ScSet1, and ScSet2 have documented histone methylation activity (Freitag, 2017). SET domain-containing HMTs in yeast are associated with vegetative growth and cell development, DNA replication, recombination and repair (Sanders et al., 2004; Kim et al., 2013). Consistent with the findings in model fungi, *AaSet1* and *AaSet2*, in addition to regulating vegetative growth, are also involved in conidiation of *A. alternata*, indicating that the function of specific SET domain-containing HMTs on cell growth and development in eukaryotes are highly conserved.

Experiments further demonstrated that both *AaSet1* and *AaSet2* are required for the resistance to Congo Red, indicating these genes are involved in the maintenance of cell wall integrity. In addition, ∆*AaHnrnp* is more sensitive to NaCl (−24.7%) and KCl (−21.9%), indicating *AaHnrnp* is required for the resistance to salts stress or osmotic stress. It is well known that many genes, which are involved in osmotic stress resistance, are required for the pathogenicity in *A. alternata*. *AaHog1*, which confers cellular resistance to salts stress, plays an essential role in fungal pathogenicity (Chung, 2013). Interestingly, *AaFus3*, which negatively regulates the resistance to osmotic stress, is also required for the fungal penetration (Lin et al., 2010). Sugar metabolism is one of the biological processes necessary for cell growth, development, and stress resistance (Ene et al., 2012). Our study revealed that ∆*AaHnrnp* and ∆*AaSet1* positively regulate the utilization of glucose, starch, and galactose. In contrast, *AaSet2* negatively regulates the utilization of sucrose and galactose, indicating the involvement of *AaSet1*, *AaSet2*, and *AaHnrnp* in carbon source metabolism in *A. alternata*. In *S. cerevisiae*, 2-Hydroxyisobutyrylation on histone H4K8 is regulated by glucose homeostasis (Huang et al., 2017). In addition, lots of DEGs of ∆*AaSet1* and ∆*AaSet2* are enriched in starch and sucrose metabolism (ko00500), galactose metabolism (ko00052), fatty acid degradation (ko00071), and so on, making it not surprising to find the disturbance of carbon source utilization. SNF1 (sucrose nonfermenting 1) is involved in nonfermentable carbon sources utilization in fungi (Alepuz et al., 1997). The *FvSnf1* gene deletion mutants of *Fusarium virguliforme* displayed serious vegetative growth defects on sole carbon source (galactose; Islam et al., 2017). In *S. cerevisiae*, SUC2 transcriptionally regulated by Snf1 is also essential for sucrose metabolism (Oezcan et al., 1997). In *A. alternata*, *AaSnf1* (AALT_g3740) is also required for the utilization of carbon source (Tang et al., 2020). However, in this study, the transcription of *AaSnf1* was not affected when *AaSet1* or *AaSet2* was knocked out, indicating that these two genes may regulate the utilization of carbon source bypassing *AaSnf1*.

Histone methylation occurs on lysine and arginine residues and is catalyzed by two families of proteins, the SET domain-containing methyltransferase family and the protein arginine methyltransferase family (Zhang and Reinberg, 2001). Biological phenotypes analysis revealed that *AaSet1* and *AaSet2* play critical in cell growth and development, indicating the SET domain-containing methyltransferases are important for various biological processes of *A. alternata*. Transcriptome analysis revealed that many DEGs are enriched in lysine biosynthesis pathway (ko00300), indicating SET domain-containing methyltransferases not only participate in the lysine methylation of histone, but also may be involved in lysine biosynthesis. Previous studies have shown that histone methylation is essential for maintaining the genome stability (Peng and Karpen, 2009; Huang et al., 2016; Mushtaq et al., 2021). We speculate that HMTs or HDMs may also are involved in maintaining genome stability. This speculation was evidenced by the result that lots of DEGs are enriched in DNA replication pathway (ko03030). In addition, the histone methylation on specific lysine also involved in DNA damage repair (Faucher and Wellinger, 2010; Gong and Miller, 2019). Our study revealed that both *AaSet2* and *AaHnrnp* are involved in the resistance to hydroxyurea (DNA-damaging agent), indicating *AaSet2* and *AaHnrnp* is required for the DNA damage repair of *A. alternata*. In addition to histone methylation, our previous revealed that histone acetylation is also required for DNA damage repair in *A. alternata* (Ma et al., 2021).

In conclusion, our study revealed that histone methylation is involved in the fungal growth, pathogenicity, conidiation, DNA damage repair, multiple stress response, and carbon source utilization of *A. alternata*. In addition, *AaSet1* and *AaSet2* are required for the transcriptional regulation of numerous genes involved in ROS detoxification, cell wall degradation, secondary metabolites biosynthesis, and RNA processing in this notorious pathogen.

## Data Availability Statement

The datasets presented in this study can be found in online repositories. The names of the repository/repositories and accession number(s) can be found at: https://www.ncbi.nlm.nih.gov/bioproject/PRJNA813997.

## Author Contributions

HM, SM, KX, and SH contributed to the conception of the study. SM, SH, SZ, YW, SD, ML, and XS performed the experiments. HM, YG, JL, and QY performed the data analysis. HM wrote the manuscript. All authors contributed to the article and approved the submitted version.

## Funding

This work was supported by the Key Project for New Agricultural Cultivar Breeding in Zhejiang Province, China (2021C02066-1) and the Major Science and Technology R&D Program of Jiangxi Province (20194ABC28007).

## Conflict of Interest

JL is employed by Natural Medicine Institute of Zhejiang YangShengTang Co., LTD.

The remaining authors declare that the research was conducted in the absence of any commercial or financial relationships that could be construed as a potential conflict of interest.

## Publisher’s Note

All claims expressed in this article are solely those of the authors and do not necessarily represent those of their affiliated organizations, or those of the publisher, the editors and the reviewers. Any product that may be evaluated in this article, or claim that may be made by its manufacturer, is not guaranteed or endorsed by the publisher.

## Footnotes

^1^ http://hme.riceblast.snu.ac.kr/
