# Supplementary material for: Histone Methylation Is Required for Virulence, Conidiation, and Multi-Stress Resistance of Alternaria alternata
Source: Front Microbiol. 2022 Jun 16;13:924476. doi: 10.3389/fmicb.2022.924476 (PMC9245015; doi:10.3389/fmicb.2022.924476)
Supplement: Supplementary file 1 [file Presentation_1.PPTX]

## Slide 1
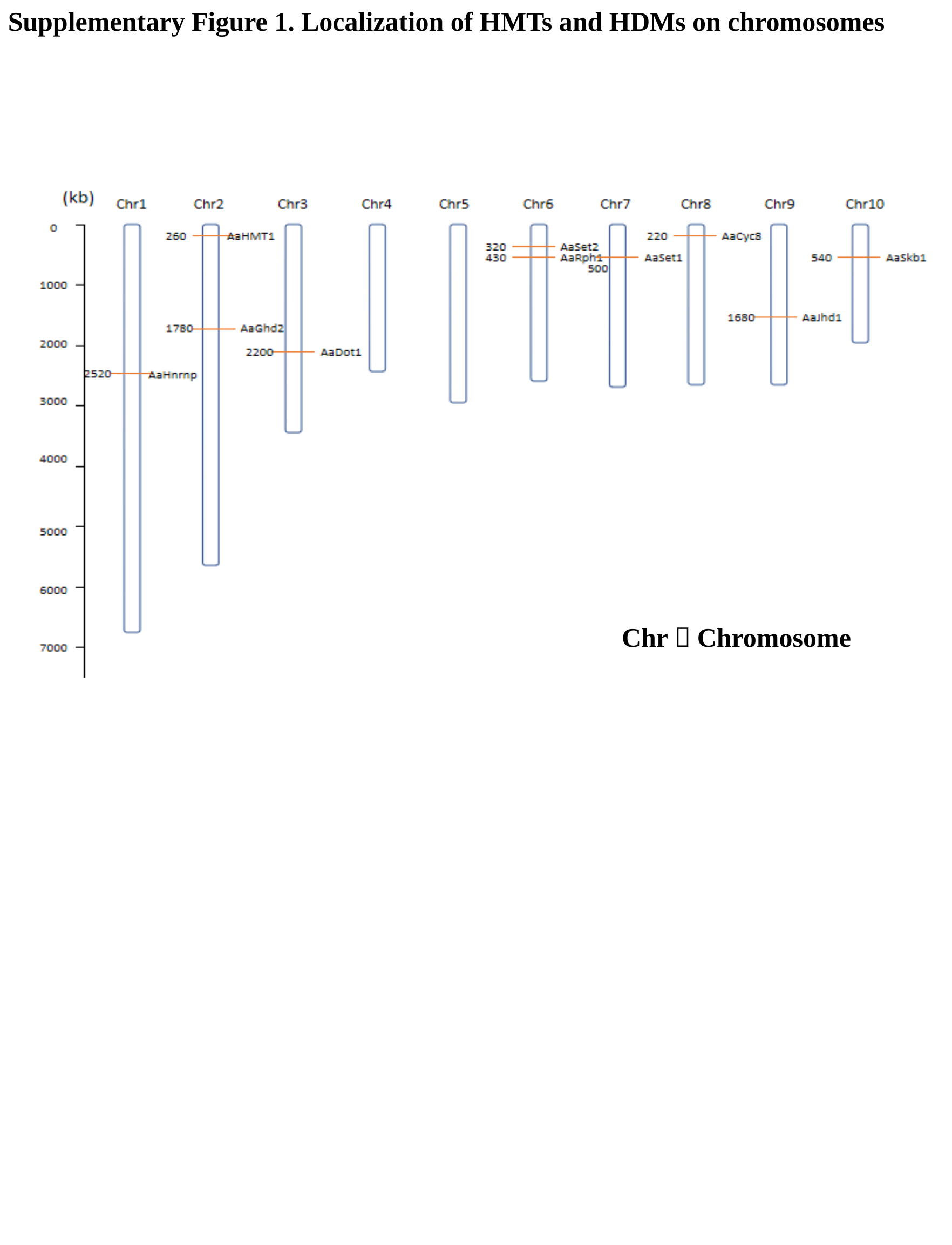

Supplementary Figure 1. Localization of HMTs and HDMs on chromosomes
Chr：Chromosome

## Slide 2
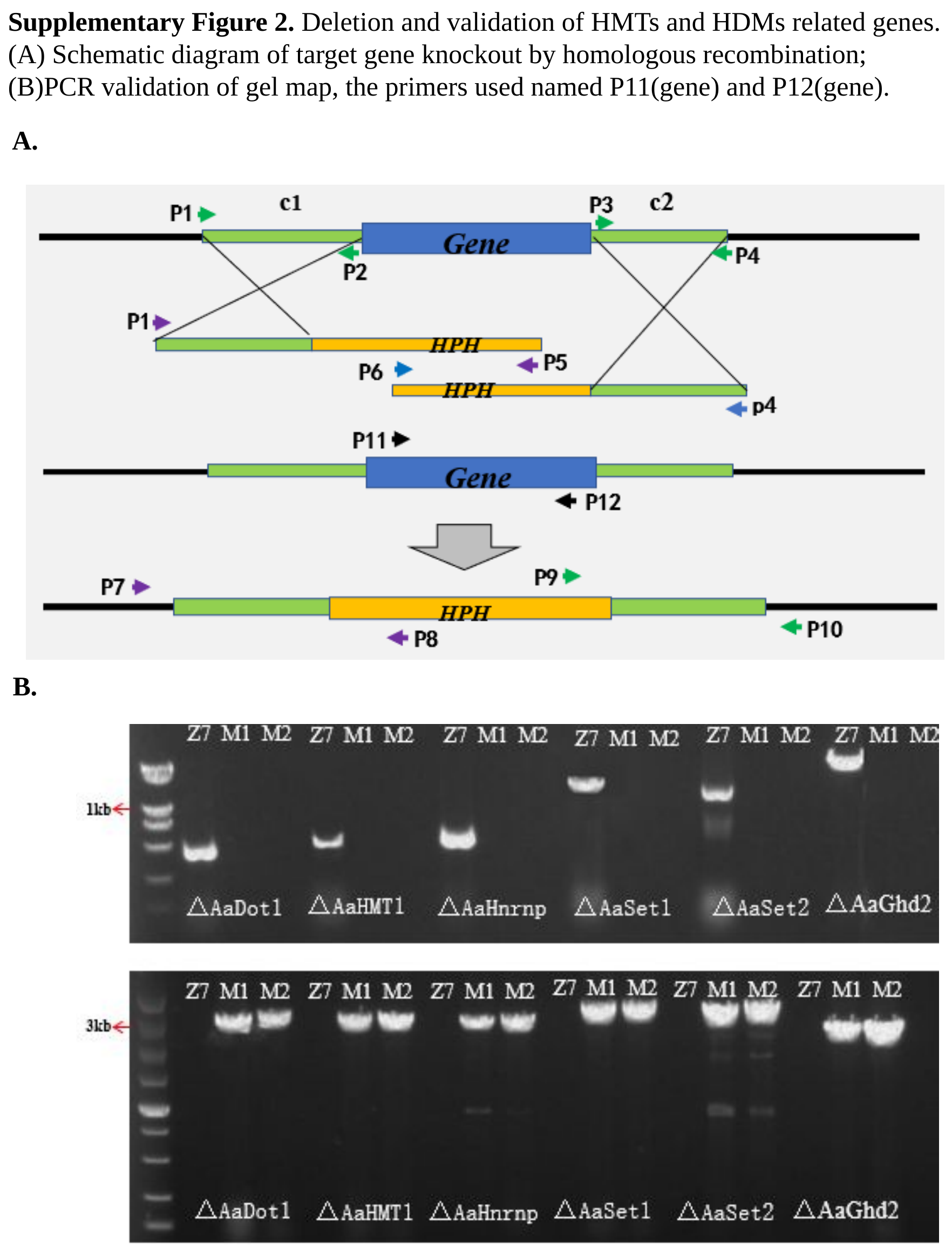

Supplementary Figure 2. Deletion and validation of HMTs and HDMs related genes. (A) Schematic diagram of target gene knockout by homologous recombination; (B)PCR validation of gel map, the primers used named P11(gene) and P12(gene).
A.
B.

## Slide 3
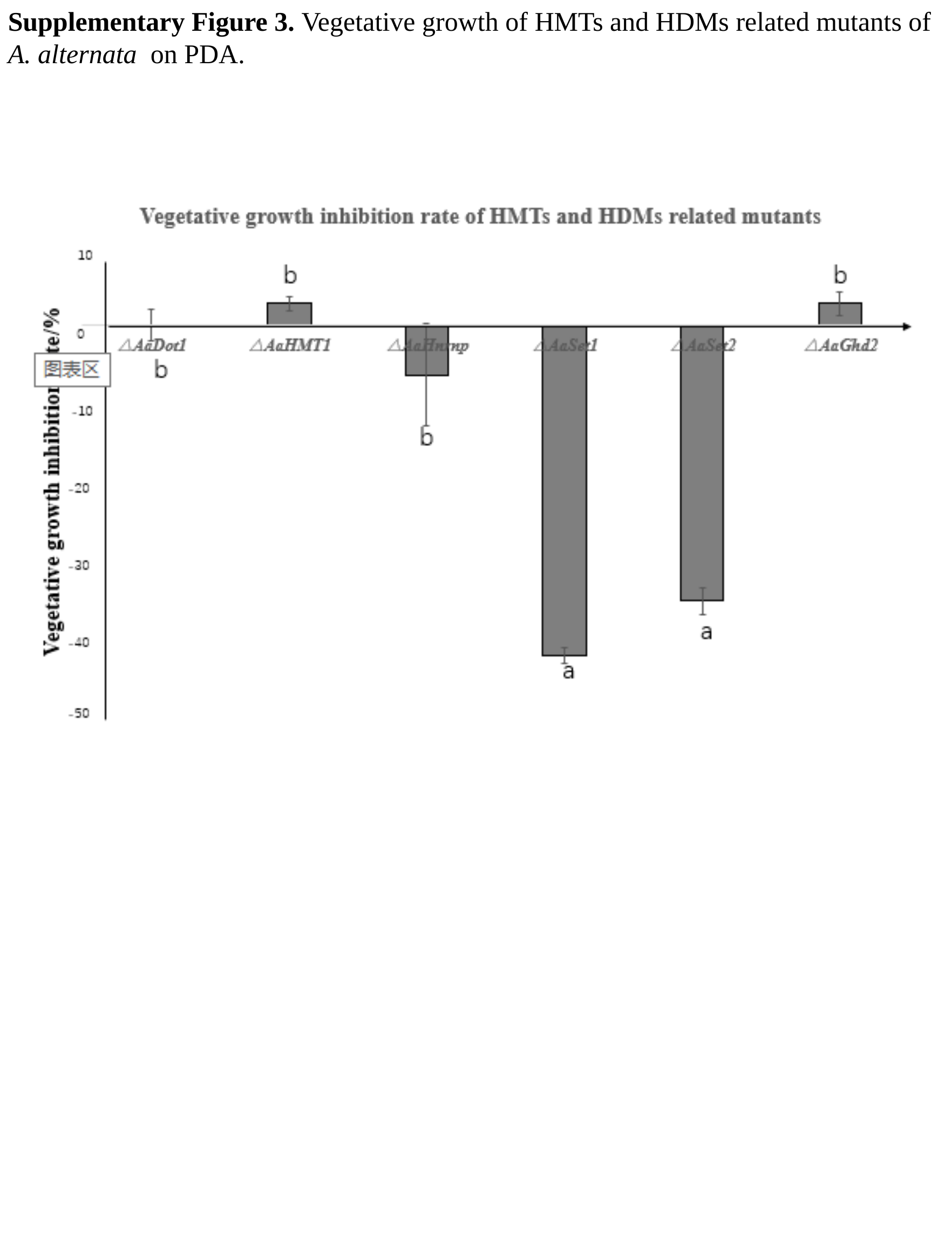

Supplementary Figure 3. Vegetative growth of HMTs and HDMs related mutants of A. alternata on PDA.

## Slide 4
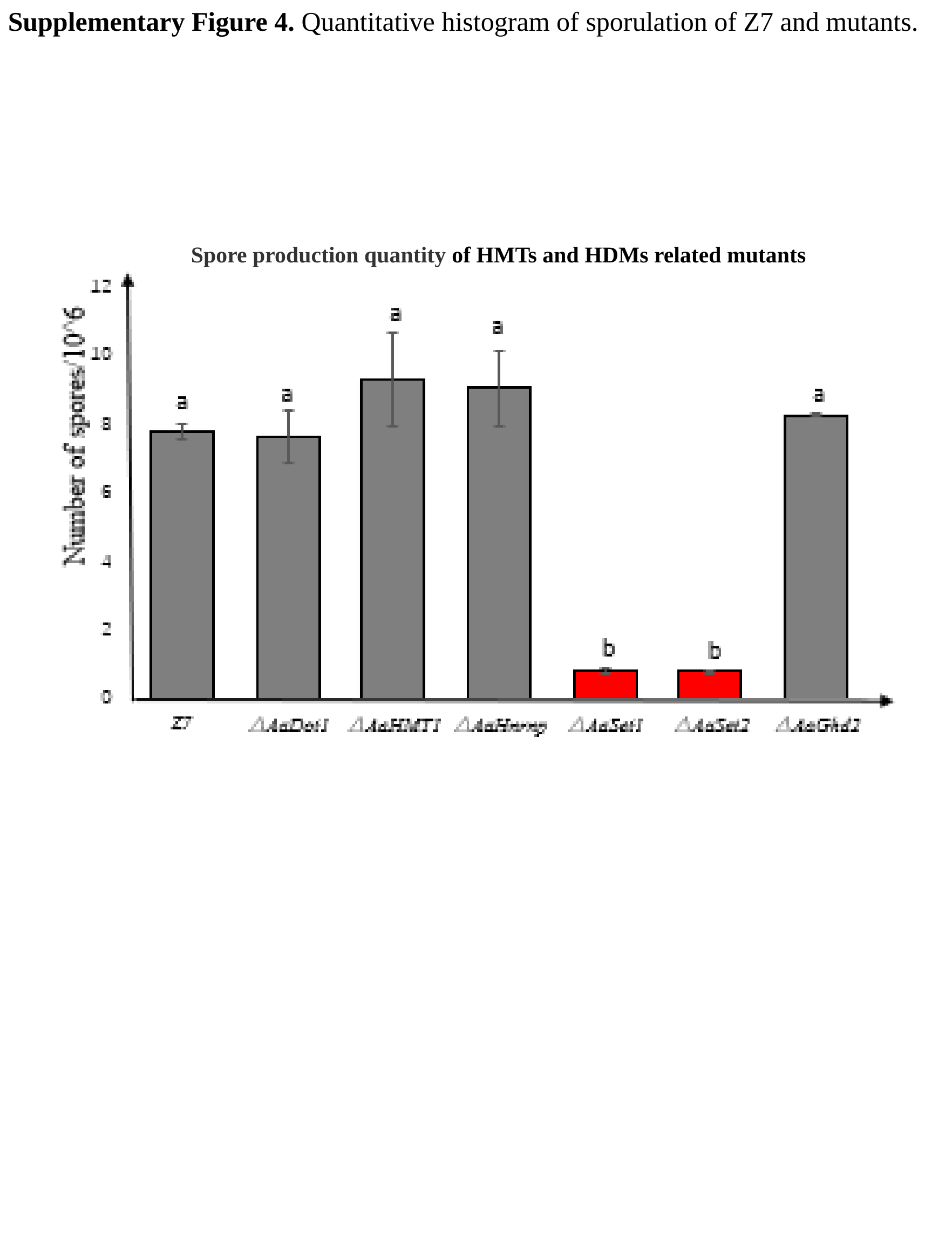

Supplementary Figure 4. Quantitative histogram of sporulation of Z7 and mutants.
Spore production quantity of HMTs and HDMs related mutants

## Slide 5
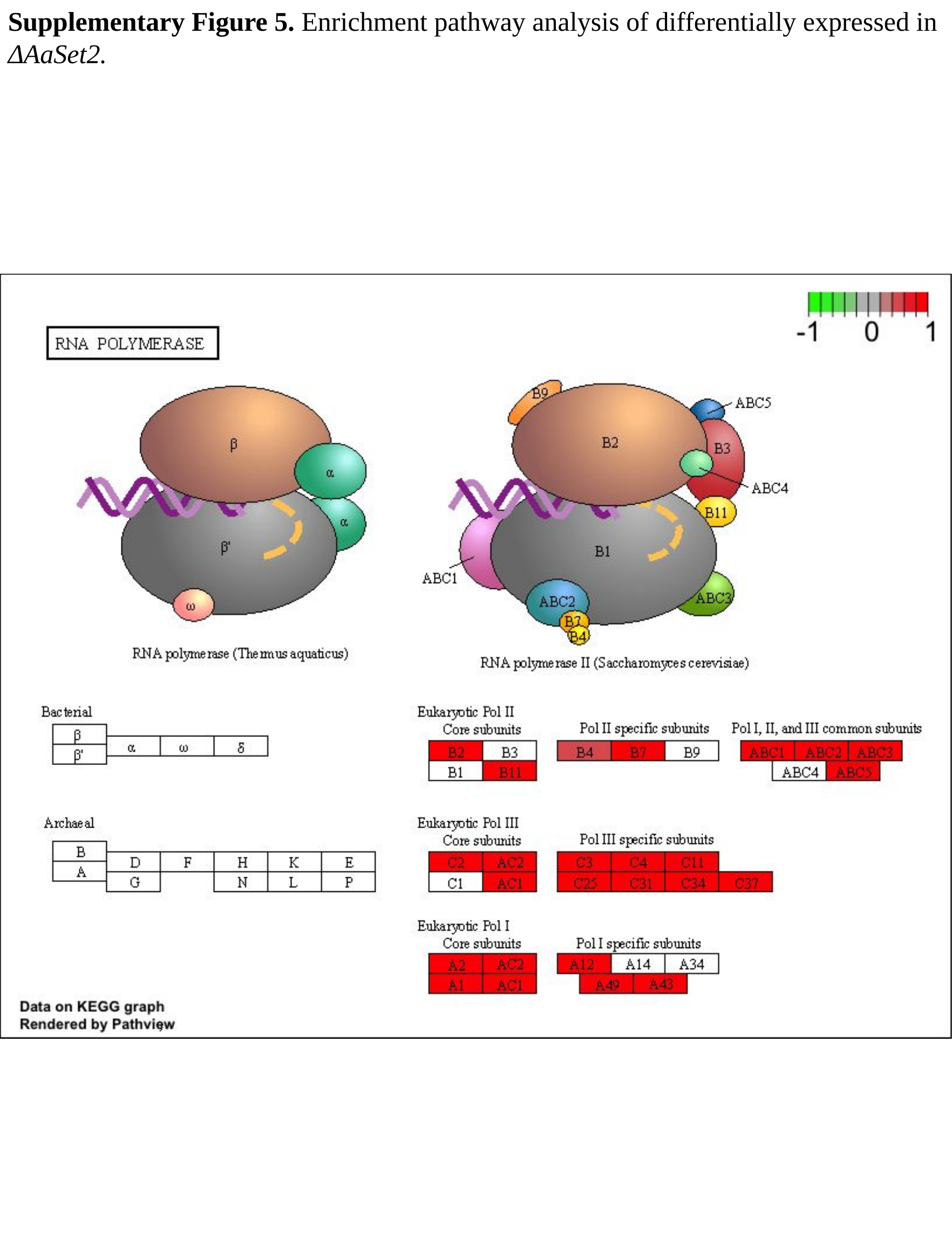

Supplementary Figure 5. Enrichment pathway analysis of differentially expressed in ΔAaSet2.

## Slide 6
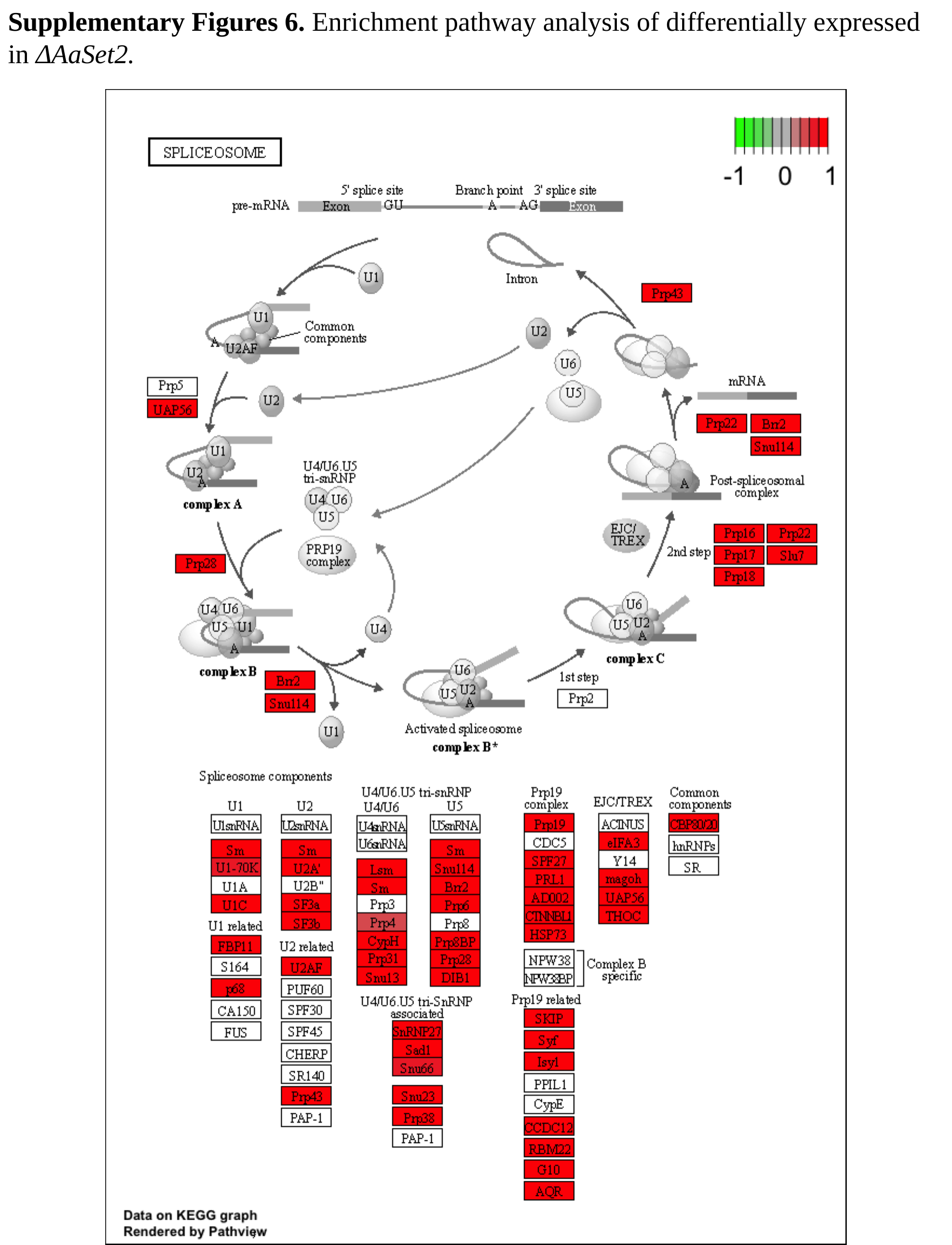

Supplementary Figures 6. Enrichment pathway analysis of differentially expressed in ΔAaSet2.

## Slide 7
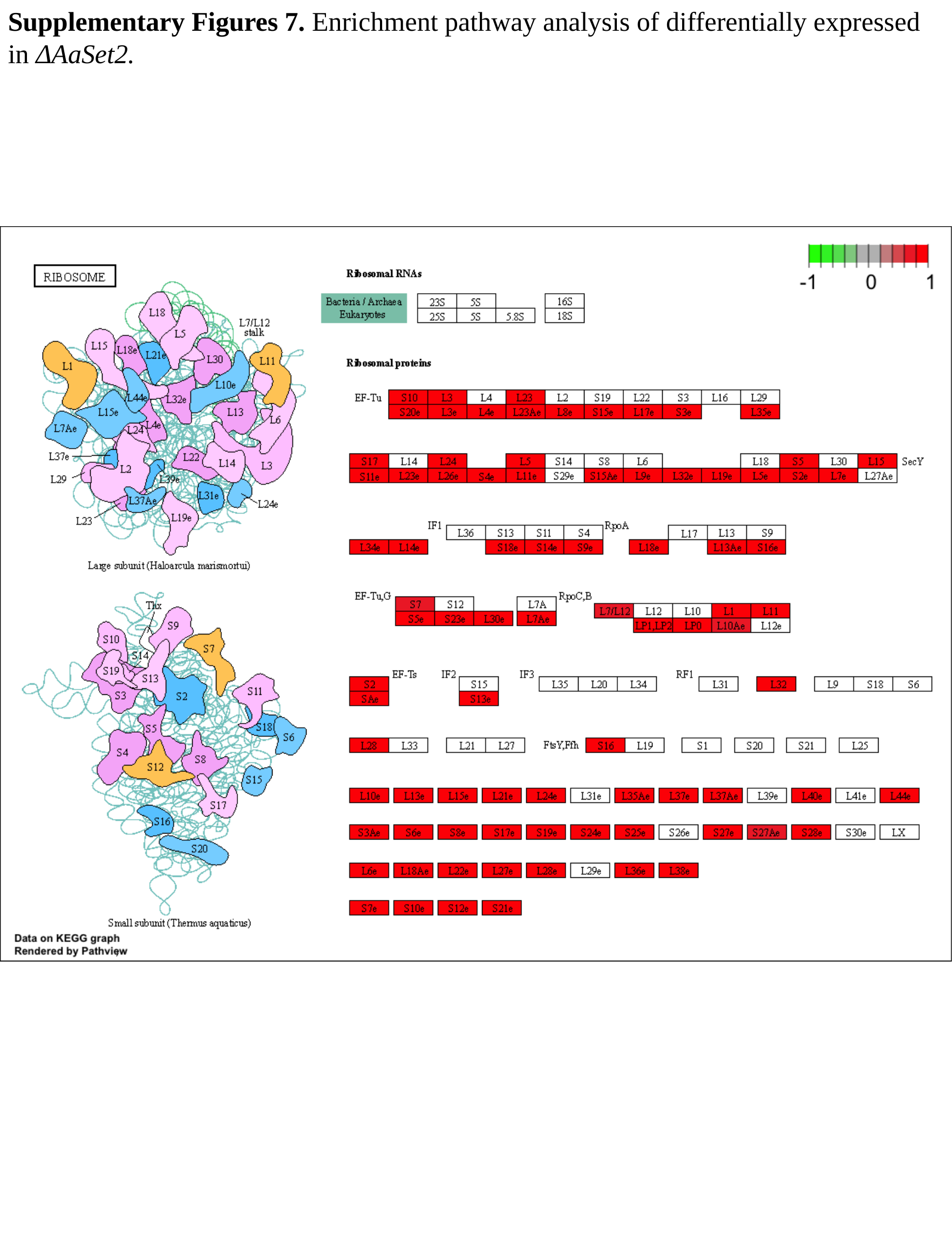

Supplementary Figures 7. Enrichment pathway analysis of differentially expressed in ΔAaSet2.

## Slide 8
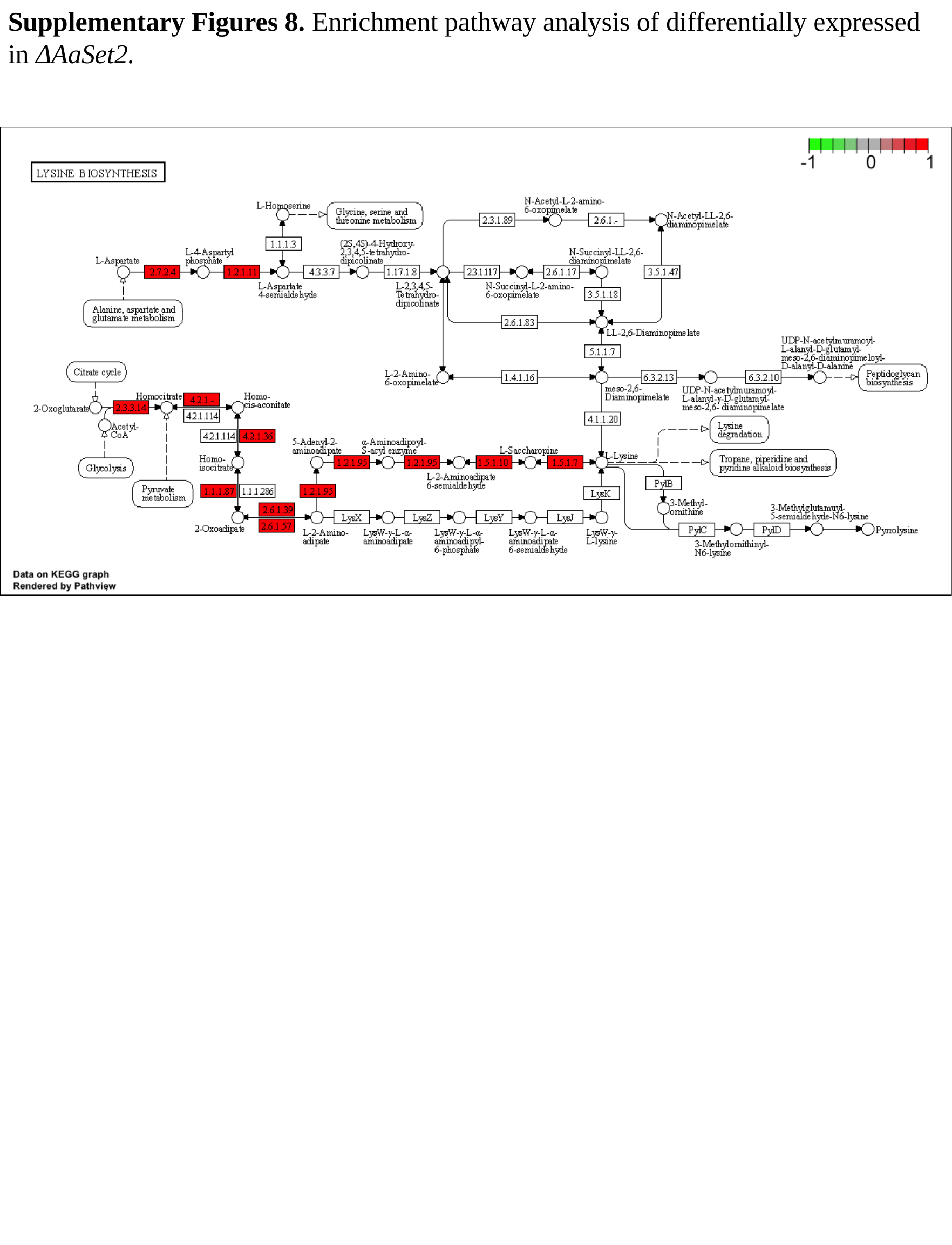

Supplementary Figures 8. Enrichment pathway analysis of differentially expressed in ΔAaSet2.

## Slide 9
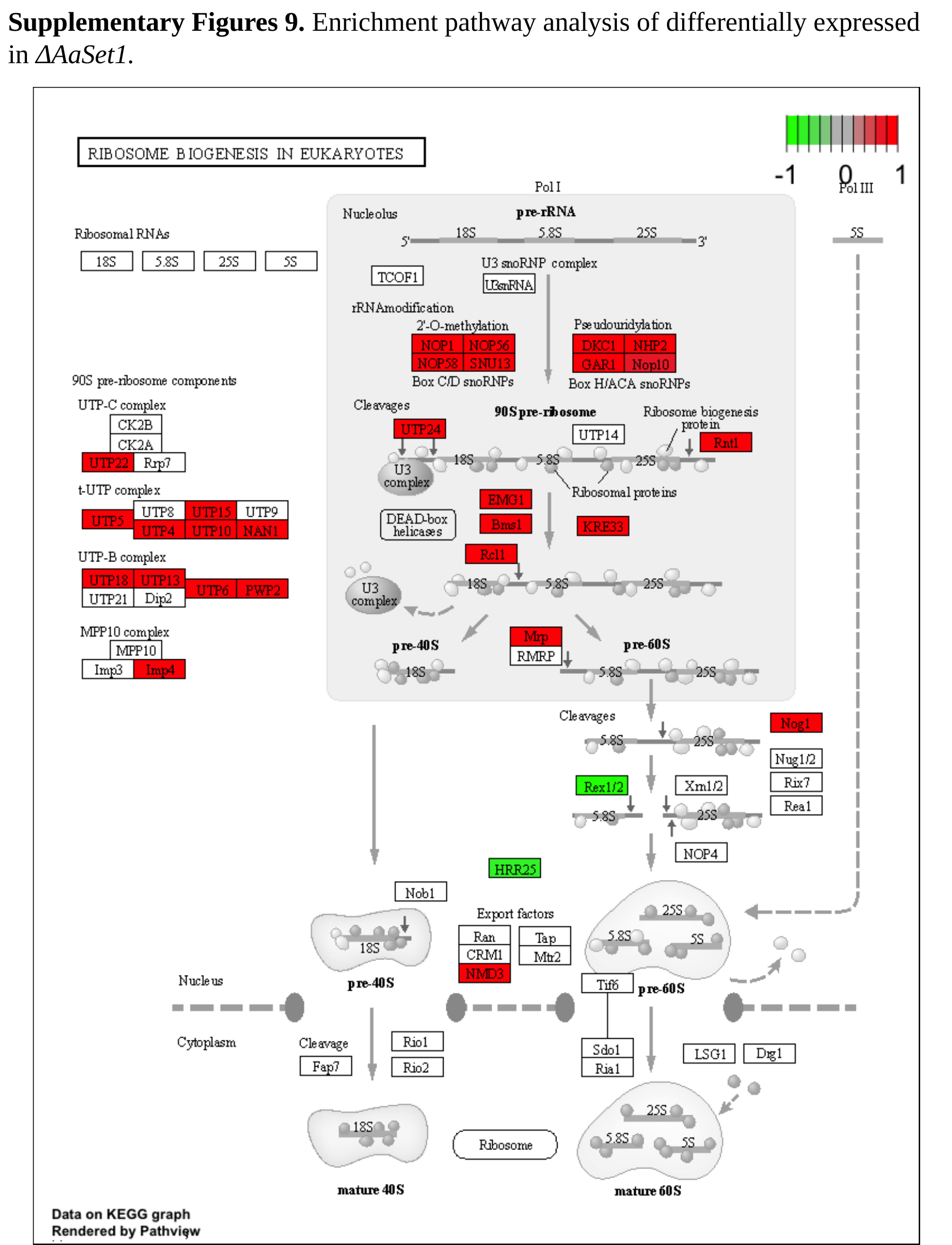

Supplementary Figures 9. Enrichment pathway analysis of differentially expressed in ΔAaSet1.

## Slide 10
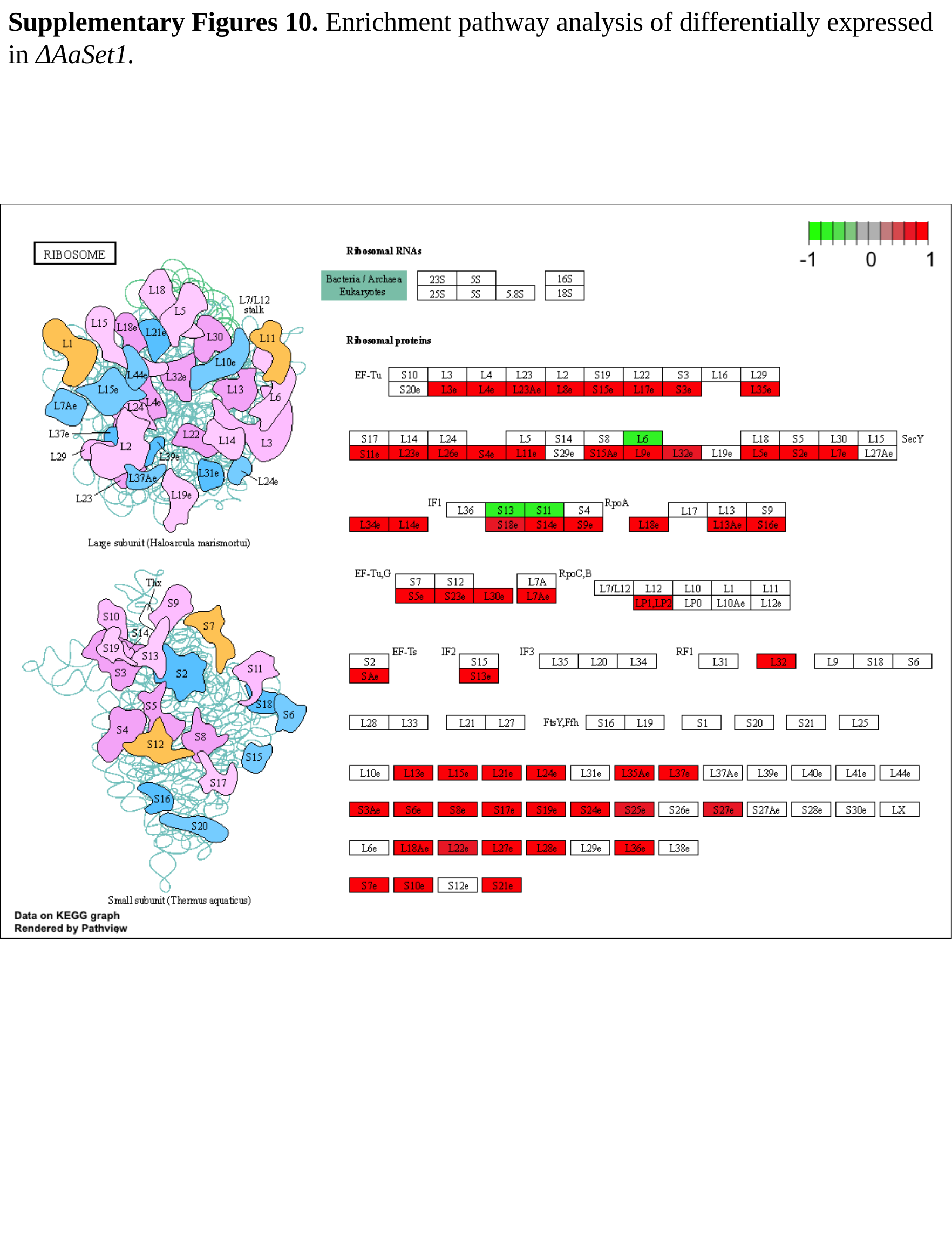

Supplementary Figures 10. Enrichment pathway analysis of differentially expressed in ΔAaSet1.

## Slide 11
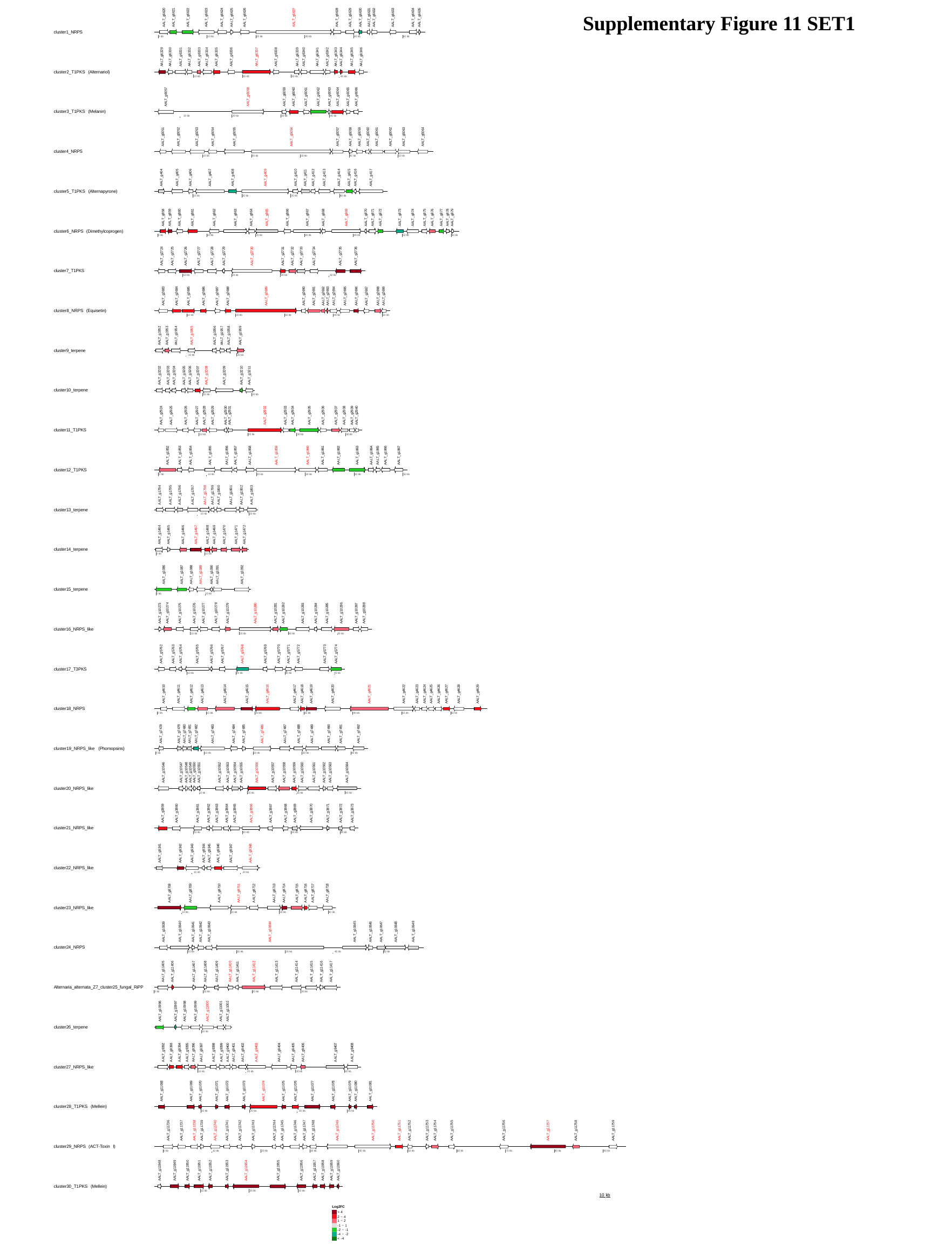

AALT_g6620
AALT_g6621
AALT_g6622
AALT_g6623
AALT_g6624
AALT_g6625
AALT_g6626
AALT_g6627
AALT_g6628
AALT_g6629
AALT_g6630
AALT_g6631
AALT_g6632
AALT_g6633
AALT_g6634
AALT_g6635
Supplementary Figure 11 SET1
cluster1_NRPS
0 kb
10 kb
20 kb
30 kb
40 kb
50 kb
AALT_g6329
AALT_g6330
AALT_g6331
AALT_g6332
AALT_g6333
AALT_g6334
AALT_g6335
AALT_g6336
AALT_g6337
AALT_g6338
AALT_g6339
AALT_g6340
AALT_g6341
AALT_g6342
AALT_g6343
AALT_g6344
AALT_g6345
AALT_g6346
cluster2_T1PKS (Alternariol)
10 kb
20 kb
30 kb
40 kb
AALT_g6057
AALT_g6058
AALT_g6059
AALT_g6060
AALT_g6061
AALT_g6062
AALT_g6063
AALT_g6064
AALT_g6065
AALT_g6066
cluster3_T1PKS (Melanin)
10 kb
20 kb
30 kb
40 kb
AALT_g5051
AALT_g5052
AALT_g5053
AALT_g5054
AALT_g5055
AALT_g5056
AALT_g5057
AALT_g5058
AALT_g5059
AALT_g5060
AALT_g5061
AALT_g5062
AALT_g5063
AALT_g5064
cluster4_NRPS
10 kb
20 kb
30 kb
40 kb
50 kb
AALT_g404
AALT_g405
AALT_g406
AALT_g407
AALT_g408
AALT_g409
AALT_g410
AALT_g411
AALT_g412
AALT_g413
AALT_g414
AALT_g415
AALT_g416
AALT_g417
cluster5_T1PKS (Alternapyrone)
10 kb
20 kb
30 kb
40 kb
AALT_g858
AALT_g859
AALT_g860
AALT_g861
AALT_g862
AALT_g863
AALT_g864
AALT_g865
AALT_g866
AALT_g867
AALT_g868
AALT_g869
AALT_g870
AALT_g871
AALT_g872
AALT_g873
AALT_g874
AALT_g875
AALT_g876
AALT_g877
AALT_g878 AALT_g879
cluster6_NRPS (Dimethylcoprogen)
0 kb
10 kb
20 kb
30 kb
40 kb
50 kb
60 kb
AALT_g2724
AALT_g2725
AALT_g2726
AALT_g2727
AALT_g2728
AALT_g2729
AALT_g2730
AALT_g2731
AALT_g2732
AALT_g2733
AALT_g2734
AALT_g2735
AALT_g2736
cluster7_T1PKS
10 kb
20 kb
30 kb
40 kb
AALT_g2683
AALT_g2684
AALT_g2685
AALT_g2686
AALT_g2687
AALT_g2688
AALT_g2689
AALT_g2690
AALT_g2691
AALT_g2692 AALT_g2693
AALT_g2694
AALT_g2695
AALT_g2696
AALT_g2697
AALT_g2698
AALT_g2699
cluster8_NRPS (Equisetin)
10 kb
20 kb
30 kb
40 kb
50 kb
AALT_g1952
AALT_g1953
AALT_g1954
AALT_g1955
AALT_g1956
AALT_g1957
AALT_g1958
AALT_g1959
cluster9_terpene
10 kb
20 kb
AALT_g3202
AALT_g3203
AALT_g3204
AALT_g3205
AALT_g3206
AALT_g3207
AALT_g3208
AALT_g3209
AALT_g3210
AALT_g3211
cluster10_terpene
10 kb
20 kb
AALT_g2924
AALT_g2925
AALT_g2926
AALT_g2927
AALT_g2928
AALT_g2929
AALT_g2930 AALT_g2931
AALT_g2932
AALT_g2933
AALT_g2934
AALT_g2935
AALT_g2936
AALT_g2937
AALT_g2938
AALT_g2939
AALT_g2940
cluster11_T1PKS
10 kb
20 kb
30 kb
40 kb
AALT_g1852
AALT_g1853
AALT_g1854
AALT_g1855
AALT_g1856
AALT_g1857
AALT_g1858
AALT_g1859
AALT_g1860
AALT_g1861
AALT_g1862
AALT_g1863
AALT_g1864
AALT_g1865
AALT_g1866
AALT_g1867
cluster12_T1PKS
0 kb
10 kb
20 kb
30 kb
40 kb
50 kb
AALT_g1794
AALT_g1795
AALT_g1796
AALT_g1797
AALT_g1798
AALT_g1799
AALT_g1800
AALT_g1801
AALT_g1802
AALT_g1803
cluster13_terpene
10 kb
20 kb
AALT_g1464
AALT_g1465
AALT_g1466
AALT_g1467
AALT_g1468
AALT_g1469
AALT_g1470
AALT_g1471
AALT_g1472
cluster14_terpene
0 kb
10 kb
AALT_g1086
AALT_g1087
AALT_g1088
AALT_g1089
AALT_g1090
AALT_g1091
AALT_g1092
cluster15_terpene
0 kb
10 kb
AALT_g10273
AALT_g10274
AALT_g10275
AALT_g10276
AALT_g10277
AALT_g10278
AALT_g10279
AALT_g10280
AALT_g10281
AALT_g10282
AALT_g10283
AALT_g10284
AALT_g10285
AALT_g10286
AALT_g10287
AALT_g10288
cluster16_NRPS_like
10 kb
20 kb
30 kb
40 kb
AALT_g5762
AALT_g5763
AALT_g5764
AALT_g5765
AALT_g5766
AALT_g5767
AALT_g5768
AALT_g5769
AALT_g5770
AALT_g5771
AALT_g5772
AALT_g5773
AALT_g5774
cluster17_T3PKS
10 kb
20 kb
30 kb
40 kb
AALT_g4610
AALT_g4611
AALT_g4612
AALT_g4613
AALT_g4614
AALT_g4615
AALT_g4616
AALT_g4617
AALT_g4618
AALT_g4619
AALT_g4620
AALT_g4621
AALT_g4622
AALT_g4623
AALT_g4624
AALT_g4625
AALT_g4626
AALT_g4627
AALT_g4628
AALT_g4629
cluster18_NRPS
0 kb
10 kb
20 kb
30 kb
40 kb
50 kb
60 kb
AALT_g7478
AALT_g7479
AALT_g7480 AALT_g7481
AALT_g7482
AALT_g7483
AALT_g7484
AALT_g7485
AALT_g7486
AALT_g7487
AALT_g7488
AALT_g7489
AALT_g7490
AALT_g7491
AALT_g7492
cluster19_NRPS_like (Phomopsins)
0 kb
10 kb
20 kb
30 kb
40 kb
AALT_g10546
AALT_g10547
AALT_g10548 AALT_g10549 AALT_g10550
AALT_g10551
AALT_g10552
AALT_g10553
AALT_g10554 AALT_g10555
AALT_g10556
AALT_g10557
AALT_g10558
AALT_g10559
AALT_g10560
AALT_g10561
AALT_g10562
AALT_g10563
AALT_g10564
cluster20_NRPS_like
10 kb
20 kb
30 kb
40 kb
AALT_g3859
AALT_g3860
AALT_g3861
AALT_g3862
AALT_g3863
AALT_g3864
AALT_g3865
AALT_g3866
AALT_g3867
AALT_g3868
AALT_g3869
AALT_g3870
AALT_g3871
AALT_g3872
AALT_g3873
cluster21_NRPS_like
10 kb
20 kb
30 kb
40 kb
AALT_g9341
AALT_g9342
AALT_g9343
AALT_g9344
AALT_g9345
AALT_g9346
AALT_g9347
AALT_g9348
cluster22_NRPS_like
10 kb
20 kb
AALT_g8708
AALT_g8709
AALT_g8710
AALT_g8711
AALT_g8712
AALT_g8713
AALT_g8714
AALT_g8715
AALT_g8716
AALT_g8717
AALT_g8718
cluster23_NRPS_like
10 kb
20 kb
30 kb
40 kb
AALT_g10639
AALT_g10640
AALT_g10641
AALT_g10642
AALT_g10643
AALT_g10644
AALT_g10645
AALT_g10646
AALT_g10647
AALT_g10648
AALT_g10649
cluster24_NRPS
10 kb
20 kb
30 kb
40 kb
50 kb
AALT_g11405
AALT_g11406
AALT_g11407
AALT_g11408
AALT_g11409
AALT_g11410
AALT_g11411
AALT_g11412
AALT_g11413
AALT_g11414
AALT_g11415
AALT_g11416
AALT_g11417
Alternaria_alternata_Z7_cluster25_fungal_RiPP
0 kb
10 kb
20 kb
30 kb
AALT_g10996
AALT_g10997
AALT_g10998
AALT_g10999
AALT_g11000
AALT_g11001
AALT_g11002
cluster26_terpene
10 kb
AALT_g9392
AALT_g9393
AALT_g9394
AALT_g9395
AALT_g9396
AALT_g9397
AALT_g9398
AALT_g9399
AALT_g9400 AALT_g9401
AALT_g9402
AALT_g9403
AALT_g9404
AALT_g9405
AALT_g9406
AALT_g9407
AALT_g9408
cluster27_NRPS_like
10 kb
20 kb
30 kb
40 kb
AALT_g11068
AALT_g11069
AALT_g11070
AALT_g11071
AALT_g11072
AALT_g11073
AALT_g11074
AALT_g11075
AALT_g11076
AALT_g11077
AALT_g11078
AALT_g11079
AALT_g11080
AALT_g11081
cluster28_T1PKS (Mellein)
10 kb
20 kb
30 kb
40 kb
AALT_g11736
AALT_g11737
AALT_g11738
AALT_g11739
AALT_g11740
AALT_g11741
AALT_g11742
AALT_g11743
AALT_g11744
AALT_g11745
AALT_g11746
AALT_g11747
AALT_g11748
AALT_g11749
AALT_g11750
AALT_g11751
AALT_g11752
AALT_g11753
AALT_g11754
AALT_g11755
AALT_g11756
AALT_g11757
AALT_g11758
AALT_g11759
cluster29_NRPS (ACT-Toxin I)
0 kb
10 kb
20 kb
30 kb
40 kb
50 kb
60 kb
70 kb
80 kb
90 kb
AALT_g11948
AALT_g11949
AALT_g11950
AALT_g11951
AALT_g11952
AALT_g11953
AALT_g11954
AALT_g11955
AALT_g11956
AALT_g11957
AALT_g11958
AALT_g11959
AALT_g11960
cluster30_T1PKS (Mellein)
10 kb
20 kb
30 kb
	10 kb
Log2FC
> 4
2 ~ 4
1 ~ 2
-1 ~ 1
-2 ~ -1
-4 ~ -2
< -4

## Slide 12
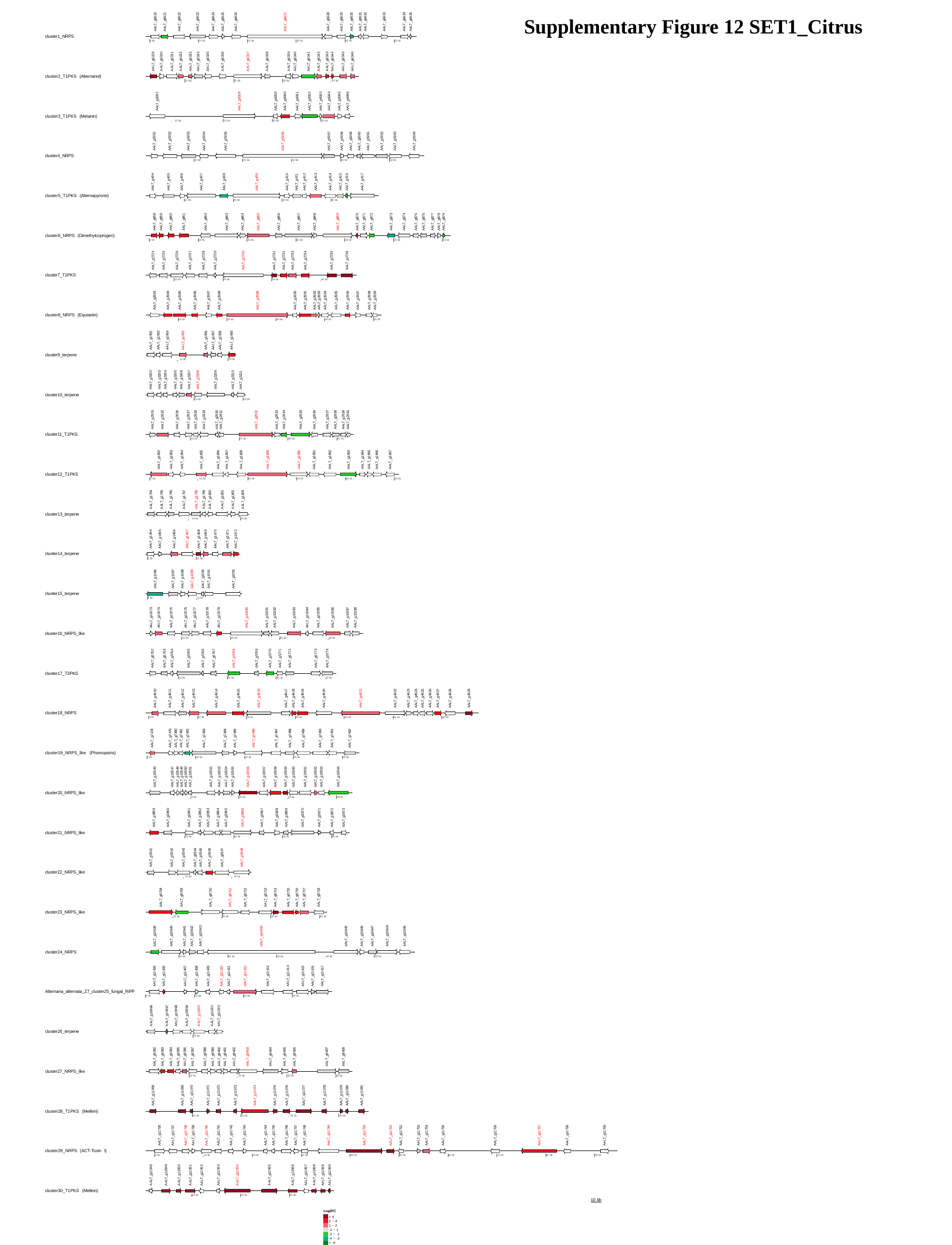

AALT_g6620
AALT_g6621
AALT_g6622
AALT_g6623
AALT_g6624
AALT_g6625
AALT_g6626
AALT_g6627
AALT_g6628
AALT_g6629
AALT_g6630
AALT_g6631
AALT_g6632
AALT_g6633
AALT_g6634
AALT_g6635
Supplementary Figure 12 SET1_Citrus
cluster1_NRPS
0 kb
10 kb
20 kb
30 kb
40 kb
50 kb
AALT_g6329
AALT_g6330
AALT_g6331
AALT_g6332
AALT_g6333
AALT_g6334
AALT_g6335
AALT_g6336
AALT_g6337
AALT_g6338
AALT_g6339
AALT_g6340
AALT_g6341
AALT_g6342
AALT_g6343
AALT_g6344
AALT_g6345
AALT_g6346
cluster2_T1PKS (Alternariol)
10 kb
20 kb
30 kb
40 kb
AALT_g6057
AALT_g6058
AALT_g6059
AALT_g6060
AALT_g6061
AALT_g6062
AALT_g6063
AALT_g6064
AALT_g6065
AALT_g6066
cluster3_T1PKS (Melanin)
10 kb
20 kb
30 kb
40 kb
AALT_g5051
AALT_g5052
AALT_g5053
AALT_g5054
AALT_g5055
AALT_g5056
AALT_g5057
AALT_g5058
AALT_g5059
AALT_g5060
AALT_g5061
AALT_g5062
AALT_g5063
AALT_g5064
cluster4_NRPS
10 kb
20 kb
30 kb
40 kb
50 kb
AALT_g404
AALT_g405
AALT_g406
AALT_g407
AALT_g408
AALT_g409
AALT_g410
AALT_g411
AALT_g412
AALT_g413
AALT_g414
AALT_g415
AALT_g416
AALT_g417
cluster5_T1PKS (Alternapyrone)
10 kb
20 kb
30 kb
40 kb
AALT_g858
AALT_g859
AALT_g860
AALT_g861
AALT_g862
AALT_g863
AALT_g864
AALT_g865
AALT_g866
AALT_g867
AALT_g868
AALT_g869
AALT_g870
AALT_g871
AALT_g872
AALT_g873
AALT_g874
AALT_g875
AALT_g876
AALT_g877
AALT_g878 AALT_g879
cluster6_NRPS (Dimethylcoprogen)
0 kb
10 kb
20 kb
30 kb
40 kb
50 kb
60 kb
AALT_g2724
AALT_g2725
AALT_g2726
AALT_g2727
AALT_g2728
AALT_g2729
AALT_g2730
AALT_g2731
AALT_g2732
AALT_g2733
AALT_g2734
AALT_g2735
AALT_g2736
cluster7_T1PKS
10 kb
20 kb
30 kb
40 kb
AALT_g2683
AALT_g2684
AALT_g2685
AALT_g2686
AALT_g2687
AALT_g2688
AALT_g2689
AALT_g2690
AALT_g2691
AALT_g2692 AALT_g2693
AALT_g2694
AALT_g2695
AALT_g2696
AALT_g2697
AALT_g2698
AALT_g2699
cluster8_NRPS (Equisetin)
10 kb
20 kb
30 kb
40 kb
50 kb
AALT_g1952
AALT_g1953
AALT_g1954
AALT_g1955
AALT_g1956
AALT_g1957
AALT_g1958
AALT_g1959
cluster9_terpene
10 kb
20 kb
AALT_g3202
AALT_g3203
AALT_g3204
AALT_g3205
AALT_g3206
AALT_g3207
AALT_g3208
AALT_g3209
AALT_g3210
AALT_g3211
cluster10_terpene
10 kb
20 kb
AALT_g2924
AALT_g2925
AALT_g2926
AALT_g2927
AALT_g2928
AALT_g2929
AALT_g2930 AALT_g2931
AALT_g2932
AALT_g2933
AALT_g2934
AALT_g2935
AALT_g2936
AALT_g2937
AALT_g2938
AALT_g2939
AALT_g2940
cluster11_T1PKS
10 kb
20 kb
30 kb
40 kb
AALT_g1852
AALT_g1853
AALT_g1854
AALT_g1855
AALT_g1856
AALT_g1857
AALT_g1858
AALT_g1859
AALT_g1860
AALT_g1861
AALT_g1862
AALT_g1863
AALT_g1864
AALT_g1865
AALT_g1866
AALT_g1867
cluster12_T1PKS
0 kb
10 kb
20 kb
30 kb
40 kb
50 kb
AALT_g1794
AALT_g1795
AALT_g1796
AALT_g1797
AALT_g1798
AALT_g1799
AALT_g1800
AALT_g1801
AALT_g1802
AALT_g1803
cluster13_terpene
10 kb
20 kb
AALT_g1464
AALT_g1465
AALT_g1466
AALT_g1467
AALT_g1468
AALT_g1469
AALT_g1470
AALT_g1471
AALT_g1472
cluster14_terpene
0 kb
10 kb
AALT_g1086
AALT_g1087
AALT_g1088
AALT_g1089
AALT_g1090
AALT_g1091
AALT_g1092
cluster15_terpene
0 kb
10 kb
AALT_g10273
AALT_g10274
AALT_g10275
AALT_g10276
AALT_g10277
AALT_g10278
AALT_g10279
AALT_g10280
AALT_g10281
AALT_g10282
AALT_g10283
AALT_g10284
AALT_g10285
AALT_g10286
AALT_g10287
AALT_g10288
cluster16_NRPS_like
10 kb
20 kb
30 kb
40 kb
AALT_g5762
AALT_g5763
AALT_g5764
AALT_g5765
AALT_g5766
AALT_g5767
AALT_g5768
AALT_g5769
AALT_g5770
AALT_g5771
AALT_g5772
AALT_g5773
AALT_g5774
cluster17_T3PKS
10 kb
20 kb
30 kb
40 kb
AALT_g4610
AALT_g4611
AALT_g4612
AALT_g4613
AALT_g4614
AALT_g4615
AALT_g4616
AALT_g4617
AALT_g4618
AALT_g4619
AALT_g4620
AALT_g4621
AALT_g4622
AALT_g4623
AALT_g4624
AALT_g4625
AALT_g4626
AALT_g4627
AALT_g4628
AALT_g4629
cluster18_NRPS
0 kb
10 kb
20 kb
30 kb
40 kb
50 kb
60 kb
AALT_g7478
AALT_g7479
AALT_g7480 AALT_g7481
AALT_g7482
AALT_g7483
AALT_g7484
AALT_g7485
AALT_g7486
AALT_g7487
AALT_g7488
AALT_g7489
AALT_g7490
AALT_g7491
AALT_g7492
cluster19_NRPS_like (Phomopsins)
0 kb
10 kb
20 kb
30 kb
40 kb
AALT_g10546
AALT_g10547
AALT_g10548 AALT_g10549 AALT_g10550
AALT_g10551
AALT_g10552
AALT_g10553
AALT_g10554 AALT_g10555
AALT_g10556
AALT_g10557
AALT_g10558
AALT_g10559
AALT_g10560
AALT_g10561
AALT_g10562
AALT_g10563
AALT_g10564
cluster20_NRPS_like
10 kb
20 kb
30 kb
40 kb
AALT_g3859
AALT_g3860
AALT_g3861
AALT_g3862
AALT_g3863
AALT_g3864
AALT_g3865
AALT_g3866
AALT_g3867
AALT_g3868
AALT_g3869
AALT_g3870
AALT_g3871
AALT_g3872
AALT_g3873
cluster21_NRPS_like
10 kb
20 kb
30 kb
40 kb
AALT_g9341
AALT_g9342
AALT_g9343
AALT_g9344
AALT_g9345
AALT_g9346
AALT_g9347
AALT_g9348
cluster22_NRPS_like
10 kb
20 kb
AALT_g8708
AALT_g8709
AALT_g8710
AALT_g8711
AALT_g8712
AALT_g8713
AALT_g8714
AALT_g8715
AALT_g8716
AALT_g8717
AALT_g8718
cluster23_NRPS_like
10 kb
20 kb
30 kb
40 kb
AALT_g10639
AALT_g10640
AALT_g10641
AALT_g10642
AALT_g10643
AALT_g10644
AALT_g10645
AALT_g10646
AALT_g10647
AALT_g10648
AALT_g10649
cluster24_NRPS
10 kb
20 kb
30 kb
40 kb
50 kb
AALT_g11405
AALT_g11406
AALT_g11407
AALT_g11408
AALT_g11409
AALT_g11410
AALT_g11411
AALT_g11412
AALT_g11413
AALT_g11414
AALT_g11415
AALT_g11416
AALT_g11417
Alternaria_alternata_Z7_cluster25_fungal_RiPP
0 kb
10 kb
20 kb
30 kb
AALT_g10996
AALT_g10997
AALT_g10998
AALT_g10999
AALT_g11000
AALT_g11001
AALT_g11002
cluster26_terpene
10 kb
AALT_g9392
AALT_g9393
AALT_g9394
AALT_g9395
AALT_g9396
AALT_g9397
AALT_g9398
AALT_g9399
AALT_g9400 AALT_g9401
AALT_g9402
AALT_g9403
AALT_g9404
AALT_g9405
AALT_g9406
AALT_g9407
AALT_g9408
cluster27_NRPS_like
10 kb
20 kb
30 kb
40 kb
AALT_g11068
AALT_g11069
AALT_g11070
AALT_g11071
AALT_g11072
AALT_g11073
AALT_g11074
AALT_g11075
AALT_g11076
AALT_g11077
AALT_g11078
AALT_g11079
AALT_g11080
AALT_g11081
cluster28_T1PKS (Mellein)
10 kb
20 kb
30 kb
40 kb
AALT_g11736
AALT_g11737
AALT_g11738
AALT_g11739
AALT_g11740
AALT_g11741
AALT_g11742
AALT_g11743
AALT_g11744
AALT_g11745
AALT_g11746
AALT_g11747
AALT_g11748
AALT_g11749
AALT_g11750
AALT_g11751
AALT_g11752
AALT_g11753
AALT_g11754
AALT_g11755
AALT_g11756
AALT_g11757
AALT_g11758
AALT_g11759
cluster29_NRPS (ACT-Toxin I)
0 kb
10 kb
20 kb
30 kb
40 kb
50 kb
60 kb
70 kb
80 kb
90 kb
AALT_g11948
AALT_g11949
AALT_g11950
AALT_g11951
AALT_g11952
AALT_g11953
AALT_g11954
AALT_g11955
AALT_g11956
AALT_g11957
AALT_g11958
AALT_g11959
AALT_g11960
cluster30_T1PKS (Mellein)
10 kb
20 kb
30 kb
	10 kb
Log2FC
> 4
2 ~ 4
1 ~ 2
-1 ~ 1
-2 ~ -1
-4 ~ -2
< -4

## Slide 13
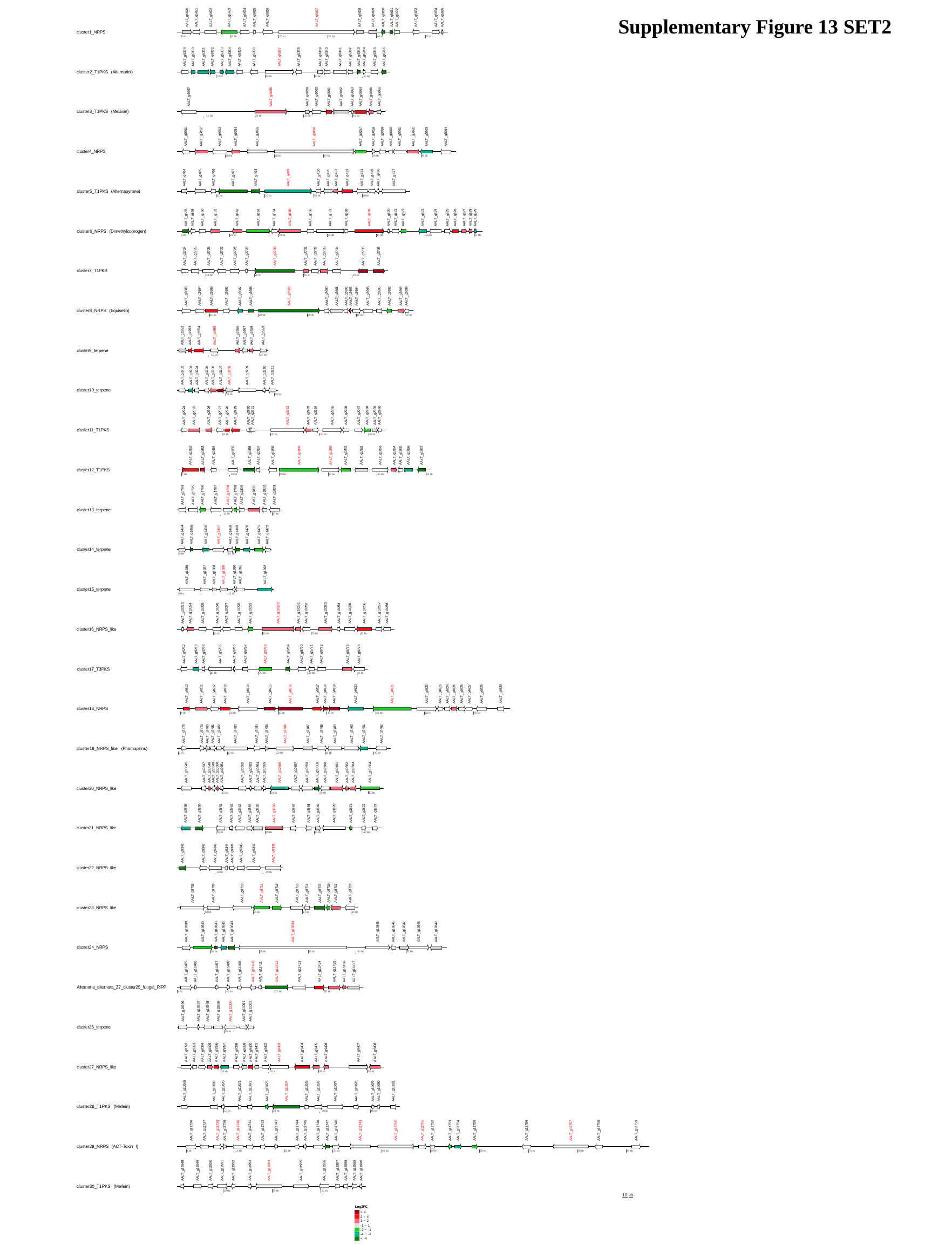

AALT_g6620
AALT_g6621
AALT_g6622
AALT_g6623
AALT_g6624
AALT_g6625
AALT_g6626
AALT_g6627
AALT_g6628
AALT_g6629
AALT_g6630
AALT_g6631
AALT_g6632
AALT_g6633
AALT_g6634
AALT_g6635
Supplementary Figure 13 SET2
cluster1_NRPS
0 kb
10 kb
20 kb
30 kb
40 kb
50 kb
AALT_g6329
AALT_g6330
AALT_g6331
AALT_g6332
AALT_g6333
AALT_g6334
AALT_g6335
AALT_g6336
AALT_g6337
AALT_g6338
AALT_g6339
AALT_g6340
AALT_g6341
AALT_g6342
AALT_g6343
AALT_g6344
AALT_g6345
AALT_g6346
cluster2_T1PKS (Alternariol)
10 kb
20 kb
30 kb
40 kb
AALT_g6057
AALT_g6058
AALT_g6059
AALT_g6060
AALT_g6061
AALT_g6062
AALT_g6063
AALT_g6064
AALT_g6065
AALT_g6066
cluster3_T1PKS (Melanin)
10 kb
20 kb
30 kb
40 kb
AALT_g5051
AALT_g5052
AALT_g5053
AALT_g5054
AALT_g5055
AALT_g5056
AALT_g5057
AALT_g5058
AALT_g5059
AALT_g5060
AALT_g5061
AALT_g5062
AALT_g5063
AALT_g5064
cluster4_NRPS
10 kb
20 kb
30 kb
40 kb
50 kb
AALT_g404
AALT_g405
AALT_g406
AALT_g407
AALT_g408
AALT_g409
AALT_g410
AALT_g411
AALT_g412
AALT_g413
AALT_g414
AALT_g415
AALT_g416
AALT_g417
cluster5_T1PKS (Alternapyrone)
10 kb
20 kb
30 kb
40 kb
AALT_g858
AALT_g859
AALT_g860
AALT_g861
AALT_g862
AALT_g863
AALT_g864
AALT_g865
AALT_g866
AALT_g867
AALT_g868
AALT_g869
AALT_g870
AALT_g871
AALT_g872
AALT_g873
AALT_g874
AALT_g875
AALT_g876
AALT_g877
AALT_g878 AALT_g879
cluster6_NRPS (Dimethylcoprogen)
0 kb
10 kb
20 kb
30 kb
40 kb
50 kb
60 kb
AALT_g2724
AALT_g2725
AALT_g2726
AALT_g2727
AALT_g2728
AALT_g2729
AALT_g2730
AALT_g2731
AALT_g2732
AALT_g2733
AALT_g2734
AALT_g2735
AALT_g2736
cluster7_T1PKS
10 kb
20 kb
30 kb
40 kb
AALT_g2683
AALT_g2684
AALT_g2685
AALT_g2686
AALT_g2687
AALT_g2688
AALT_g2689
AALT_g2690
AALT_g2691
AALT_g2692 AALT_g2693
AALT_g2694
AALT_g2695
AALT_g2696
AALT_g2697
AALT_g2698
AALT_g2699
cluster8_NRPS (Equisetin)
10 kb
20 kb
30 kb
40 kb
50 kb
AALT_g1952
AALT_g1953
AALT_g1954
AALT_g1955
AALT_g1956
AALT_g1957
AALT_g1958
AALT_g1959
cluster9_terpene
10 kb
20 kb
AALT_g3202
AALT_g3203
AALT_g3204
AALT_g3205
AALT_g3206
AALT_g3207
AALT_g3208
AALT_g3209
AALT_g3210
AALT_g3211
cluster10_terpene
10 kb
20 kb
AALT_g2924
AALT_g2925
AALT_g2926
AALT_g2927
AALT_g2928
AALT_g2929
AALT_g2930 AALT_g2931
AALT_g2932
AALT_g2933
AALT_g2934
AALT_g2935
AALT_g2936
AALT_g2937
AALT_g2938
AALT_g2939
AALT_g2940
cluster11_T1PKS
10 kb
20 kb
30 kb
40 kb
AALT_g1852
AALT_g1853
AALT_g1854
AALT_g1855
AALT_g1856
AALT_g1857
AALT_g1858
AALT_g1859
AALT_g1860
AALT_g1861
AALT_g1862
AALT_g1863
AALT_g1864
AALT_g1865
AALT_g1866
AALT_g1867
cluster12_T1PKS
0 kb
10 kb
20 kb
30 kb
40 kb
50 kb
AALT_g1794
AALT_g1795
AALT_g1796
AALT_g1797
AALT_g1798
AALT_g1799
AALT_g1800
AALT_g1801
AALT_g1802
AALT_g1803
cluster13_terpene
10 kb
20 kb
AALT_g1464
AALT_g1465
AALT_g1466
AALT_g1467
AALT_g1468
AALT_g1469
AALT_g1470
AALT_g1471
AALT_g1472
cluster14_terpene
0 kb
10 kb
AALT_g1086
AALT_g1087
AALT_g1088
AALT_g1089
AALT_g1090
AALT_g1091
AALT_g1092
cluster15_terpene
0 kb
10 kb
AALT_g10273
AALT_g10274
AALT_g10275
AALT_g10276
AALT_g10277
AALT_g10278
AALT_g10279
AALT_g10280
AALT_g10281
AALT_g10282
AALT_g10283
AALT_g10284
AALT_g10285
AALT_g10286
AALT_g10287
AALT_g10288
cluster16_NRPS_like
10 kb
20 kb
30 kb
40 kb
AALT_g5762
AALT_g5763
AALT_g5764
AALT_g5765
AALT_g5766
AALT_g5767
AALT_g5768
AALT_g5769
AALT_g5770
AALT_g5771
AALT_g5772
AALT_g5773
AALT_g5774
cluster17_T3PKS
10 kb
20 kb
30 kb
40 kb
AALT_g4610
AALT_g4611
AALT_g4612
AALT_g4613
AALT_g4614
AALT_g4615
AALT_g4616
AALT_g4617
AALT_g4618
AALT_g4619
AALT_g4620
AALT_g4621
AALT_g4622
AALT_g4623
AALT_g4624
AALT_g4625
AALT_g4626
AALT_g4627
AALT_g4628
AALT_g4629
cluster18_NRPS
0 kb
10 kb
20 kb
30 kb
40 kb
50 kb
60 kb
AALT_g7478
AALT_g7479
AALT_g7480 AALT_g7481
AALT_g7482
AALT_g7483
AALT_g7484
AALT_g7485
AALT_g7486
AALT_g7487
AALT_g7488
AALT_g7489
AALT_g7490
AALT_g7491
AALT_g7492
cluster19_NRPS_like (Phomopsins)
0 kb
10 kb
20 kb
30 kb
40 kb
AALT_g10546
AALT_g10547
AALT_g10548 AALT_g10549 AALT_g10550
AALT_g10551
AALT_g10552
AALT_g10553
AALT_g10554 AALT_g10555
AALT_g10556
AALT_g10557
AALT_g10558
AALT_g10559
AALT_g10560
AALT_g10561
AALT_g10562
AALT_g10563
AALT_g10564
cluster20_NRPS_like
10 kb
20 kb
30 kb
40 kb
AALT_g3859
AALT_g3860
AALT_g3861
AALT_g3862
AALT_g3863
AALT_g3864
AALT_g3865
AALT_g3866
AALT_g3867
AALT_g3868
AALT_g3869
AALT_g3870
AALT_g3871
AALT_g3872
AALT_g3873
cluster21_NRPS_like
10 kb
20 kb
30 kb
40 kb
AALT_g9341
AALT_g9342
AALT_g9343
AALT_g9344
AALT_g9345
AALT_g9346
AALT_g9347
AALT_g9348
cluster22_NRPS_like
10 kb
20 kb
AALT_g8708
AALT_g8709
AALT_g8710
AALT_g8711
AALT_g8712
AALT_g8713
AALT_g8714
AALT_g8715
AALT_g8716
AALT_g8717
AALT_g8718
cluster23_NRPS_like
10 kb
20 kb
30 kb
40 kb
AALT_g10639
AALT_g10640
AALT_g10641
AALT_g10642
AALT_g10643
AALT_g10644
AALT_g10645
AALT_g10646
AALT_g10647
AALT_g10648
AALT_g10649
cluster24_NRPS
10 kb
20 kb
30 kb
40 kb
50 kb
AALT_g11405
AALT_g11406
AALT_g11407
AALT_g11408
AALT_g11409
AALT_g11410
AALT_g11411
AALT_g11412
AALT_g11413
AALT_g11414
AALT_g11415
AALT_g11416
AALT_g11417
Alternaria_alternata_Z7_cluster25_fungal_RiPP
0 kb
10 kb
20 kb
30 kb
AALT_g10996
AALT_g10997
AALT_g10998
AALT_g10999
AALT_g11000
AALT_g11001
AALT_g11002
cluster26_terpene
10 kb
AALT_g9392
AALT_g9393
AALT_g9394
AALT_g9395
AALT_g9396
AALT_g9397
AALT_g9398
AALT_g9399
AALT_g9400 AALT_g9401
AALT_g9402
AALT_g9403
AALT_g9404
AALT_g9405
AALT_g9406
AALT_g9407
AALT_g9408
cluster27_NRPS_like
10 kb
20 kb
30 kb
40 kb
AALT_g11068
AALT_g11069
AALT_g11070
AALT_g11071
AALT_g11072
AALT_g11073
AALT_g11074
AALT_g11075
AALT_g11076
AALT_g11077
AALT_g11078
AALT_g11079
AALT_g11080
AALT_g11081
cluster28_T1PKS (Mellein)
10 kb
20 kb
30 kb
40 kb
AALT_g11736
AALT_g11737
AALT_g11738
AALT_g11739
AALT_g11740
AALT_g11741
AALT_g11742
AALT_g11743
AALT_g11744
AALT_g11745
AALT_g11746
AALT_g11747
AALT_g11748
AALT_g11749
AALT_g11750
AALT_g11751
AALT_g11752
AALT_g11753
AALT_g11754
AALT_g11755
AALT_g11756
AALT_g11757
AALT_g11758
AALT_g11759
cluster29_NRPS (ACT-Toxin I)
0 kb
10 kb
20 kb
30 kb
40 kb
50 kb
60 kb
70 kb
80 kb
90 kb
AALT_g11948
AALT_g11949
AALT_g11950
AALT_g11951
AALT_g11952
AALT_g11953
AALT_g11954
AALT_g11955
AALT_g11956
AALT_g11957
AALT_g11958
AALT_g11959
AALT_g11960
cluster30_T1PKS (Mellein)
10 kb
20 kb
30 kb
	10 kb
Log2FC
> 4
2 ~ 4
1 ~ 2
-1 ~ 1
-2 ~ -1
-4 ~ -2
< -4

## Slide 14
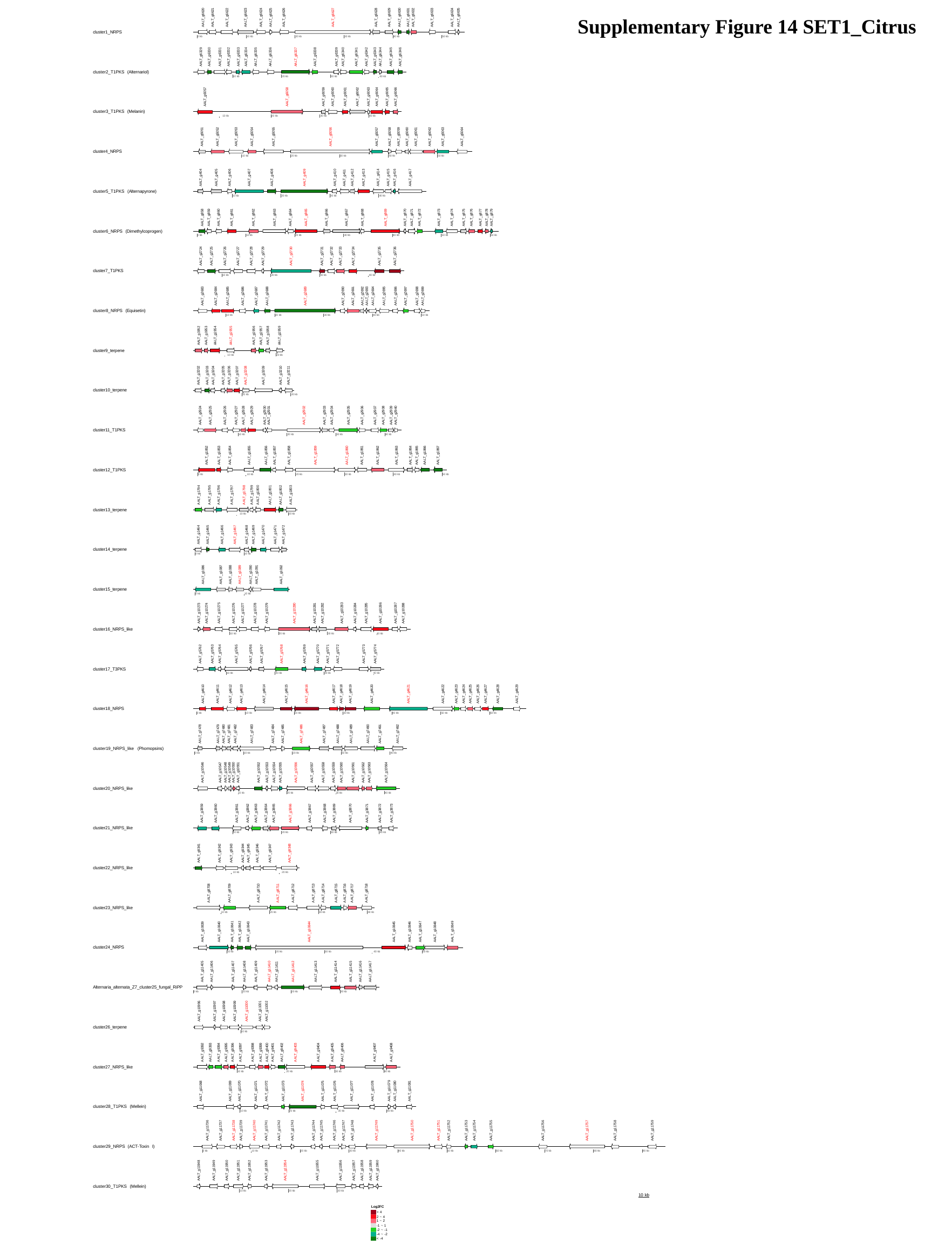

AALT_g6620
AALT_g6621
AALT_g6622
AALT_g6623
AALT_g6624
AALT_g6625
AALT_g6626
AALT_g6627
AALT_g6628
AALT_g6629
AALT_g6630
AALT_g6631
AALT_g6632
AALT_g6633
AALT_g6634
AALT_g6635
Supplementary Figure 14 SET1_Citrus
cluster1_NRPS
0 kb
10 kb
20 kb
30 kb
40 kb
50 kb
AALT_g6329
AALT_g6330
AALT_g6331
AALT_g6332
AALT_g6333
AALT_g6334
AALT_g6335
AALT_g6336
AALT_g6337
AALT_g6338
AALT_g6339
AALT_g6340
AALT_g6341
AALT_g6342
AALT_g6343
AALT_g6344
AALT_g6345
AALT_g6346
cluster2_T1PKS (Alternariol)
10 kb
20 kb
30 kb
40 kb
AALT_g6057
AALT_g6058
AALT_g6059
AALT_g6060
AALT_g6061
AALT_g6062
AALT_g6063
AALT_g6064
AALT_g6065
AALT_g6066
cluster3_T1PKS (Melanin)
10 kb
20 kb
30 kb
40 kb
AALT_g5051
AALT_g5052
AALT_g5053
AALT_g5054
AALT_g5055
AALT_g5056
AALT_g5057
AALT_g5058
AALT_g5059
AALT_g5060
AALT_g5061
AALT_g5062
AALT_g5063
AALT_g5064
cluster4_NRPS
10 kb
20 kb
30 kb
40 kb
50 kb
AALT_g404
AALT_g405
AALT_g406
AALT_g407
AALT_g408
AALT_g409
AALT_g410
AALT_g411
AALT_g412
AALT_g413
AALT_g414
AALT_g415
AALT_g416
AALT_g417
cluster5_T1PKS (Alternapyrone)
10 kb
20 kb
30 kb
40 kb
AALT_g858
AALT_g859
AALT_g860
AALT_g861
AALT_g862
AALT_g863
AALT_g864
AALT_g865
AALT_g866
AALT_g867
AALT_g868
AALT_g869
AALT_g870
AALT_g871
AALT_g872
AALT_g873
AALT_g874
AALT_g875
AALT_g876
AALT_g877
AALT_g878 AALT_g879
cluster6_NRPS (Dimethylcoprogen)
0 kb
10 kb
20 kb
30 kb
40 kb
50 kb
60 kb
AALT_g2724
AALT_g2725
AALT_g2726
AALT_g2727
AALT_g2728
AALT_g2729
AALT_g2730
AALT_g2731
AALT_g2732
AALT_g2733
AALT_g2734
AALT_g2735
AALT_g2736
cluster7_T1PKS
10 kb
20 kb
30 kb
40 kb
AALT_g2683
AALT_g2684
AALT_g2685
AALT_g2686
AALT_g2687
AALT_g2688
AALT_g2689
AALT_g2690
AALT_g2691
AALT_g2692 AALT_g2693
AALT_g2694
AALT_g2695
AALT_g2696
AALT_g2697
AALT_g2698
AALT_g2699
cluster8_NRPS (Equisetin)
10 kb
20 kb
30 kb
40 kb
50 kb
AALT_g1952
AALT_g1953
AALT_g1954
AALT_g1955
AALT_g1956
AALT_g1957
AALT_g1958
AALT_g1959
cluster9_terpene
10 kb
20 kb
AALT_g3202
AALT_g3203
AALT_g3204
AALT_g3205
AALT_g3206
AALT_g3207
AALT_g3208
AALT_g3209
AALT_g3210
AALT_g3211
cluster10_terpene
10 kb
20 kb
AALT_g2924
AALT_g2925
AALT_g2926
AALT_g2927
AALT_g2928
AALT_g2929
AALT_g2930 AALT_g2931
AALT_g2932
AALT_g2933
AALT_g2934
AALT_g2935
AALT_g2936
AALT_g2937
AALT_g2938
AALT_g2939
AALT_g2940
cluster11_T1PKS
10 kb
20 kb
30 kb
40 kb
AALT_g1852
AALT_g1853
AALT_g1854
AALT_g1855
AALT_g1856
AALT_g1857
AALT_g1858
AALT_g1859
AALT_g1860
AALT_g1861
AALT_g1862
AALT_g1863
AALT_g1864
AALT_g1865
AALT_g1866
AALT_g1867
cluster12_T1PKS
0 kb
10 kb
20 kb
30 kb
40 kb
50 kb
AALT_g1794
AALT_g1795
AALT_g1796
AALT_g1797
AALT_g1798
AALT_g1799
AALT_g1800
AALT_g1801
AALT_g1802
AALT_g1803
cluster13_terpene
10 kb
20 kb
AALT_g1464
AALT_g1465
AALT_g1466
AALT_g1467
AALT_g1468
AALT_g1469
AALT_g1470
AALT_g1471
AALT_g1472
cluster14_terpene
0 kb
10 kb
AALT_g1086
AALT_g1087
AALT_g1088
AALT_g1089
AALT_g1090
AALT_g1091
AALT_g1092
cluster15_terpene
0 kb
10 kb
AALT_g10273
AALT_g10274
AALT_g10275
AALT_g10276
AALT_g10277
AALT_g10278
AALT_g10279
AALT_g10280
AALT_g10281
AALT_g10282
AALT_g10283
AALT_g10284
AALT_g10285
AALT_g10286
AALT_g10287
AALT_g10288
cluster16_NRPS_like
10 kb
20 kb
30 kb
40 kb
AALT_g5762
AALT_g5763
AALT_g5764
AALT_g5765
AALT_g5766
AALT_g5767
AALT_g5768
AALT_g5769
AALT_g5770
AALT_g5771
AALT_g5772
AALT_g5773
AALT_g5774
cluster17_T3PKS
10 kb
20 kb
30 kb
40 kb
AALT_g4610
AALT_g4611
AALT_g4612
AALT_g4613
AALT_g4614
AALT_g4615
AALT_g4616
AALT_g4617
AALT_g4618
AALT_g4619
AALT_g4620
AALT_g4621
AALT_g4622
AALT_g4623
AALT_g4624
AALT_g4625
AALT_g4626
AALT_g4627
AALT_g4628
AALT_g4629
cluster18_NRPS
0 kb
10 kb
20 kb
30 kb
40 kb
50 kb
60 kb
AALT_g7478
AALT_g7479
AALT_g7480 AALT_g7481
AALT_g7482
AALT_g7483
AALT_g7484
AALT_g7485
AALT_g7486
AALT_g7487
AALT_g7488
AALT_g7489
AALT_g7490
AALT_g7491
AALT_g7492
cluster19_NRPS_like (Phomopsins)
0 kb
10 kb
20 kb
30 kb
40 kb
AALT_g10546
AALT_g10547
AALT_g10548 AALT_g10549 AALT_g10550
AALT_g10551
AALT_g10552
AALT_g10553
AALT_g10554 AALT_g10555
AALT_g10556
AALT_g10557
AALT_g10558
AALT_g10559
AALT_g10560
AALT_g10561
AALT_g10562
AALT_g10563
AALT_g10564
cluster20_NRPS_like
10 kb
20 kb
30 kb
40 kb
AALT_g3859
AALT_g3860
AALT_g3861
AALT_g3862
AALT_g3863
AALT_g3864
AALT_g3865
AALT_g3866
AALT_g3867
AALT_g3868
AALT_g3869
AALT_g3870
AALT_g3871
AALT_g3872
AALT_g3873
cluster21_NRPS_like
10 kb
20 kb
30 kb
40 kb
AALT_g9341
AALT_g9342
AALT_g9343
AALT_g9344
AALT_g9345
AALT_g9346
AALT_g9347
AALT_g9348
cluster22_NRPS_like
10 kb
20 kb
AALT_g8708
AALT_g8709
AALT_g8710
AALT_g8711
AALT_g8712
AALT_g8713
AALT_g8714
AALT_g8715
AALT_g8716
AALT_g8717
AALT_g8718
cluster23_NRPS_like
10 kb
20 kb
30 kb
40 kb
AALT_g10639
AALT_g10640
AALT_g10641
AALT_g10642
AALT_g10643
AALT_g10644
AALT_g10645
AALT_g10646
AALT_g10647
AALT_g10648
AALT_g10649
cluster24_NRPS
10 kb
20 kb
30 kb
40 kb
50 kb
AALT_g11405
AALT_g11406
AALT_g11407
AALT_g11408
AALT_g11409
AALT_g11410
AALT_g11411
AALT_g11412
AALT_g11413
AALT_g11414
AALT_g11415
AALT_g11416
AALT_g11417
Alternaria_alternata_Z7_cluster25_fungal_RiPP
0 kb
10 kb
20 kb
30 kb
AALT_g10996
AALT_g10997
AALT_g10998
AALT_g10999
AALT_g11000
AALT_g11001
AALT_g11002
cluster26_terpene
10 kb
AALT_g9392
AALT_g9393
AALT_g9394
AALT_g9395
AALT_g9396
AALT_g9397
AALT_g9398
AALT_g9399
AALT_g9400 AALT_g9401
AALT_g9402
AALT_g9403
AALT_g9404
AALT_g9405
AALT_g9406
AALT_g9407
AALT_g9408
cluster27_NRPS_like
10 kb
20 kb
30 kb
40 kb
AALT_g11068
AALT_g11069
AALT_g11070
AALT_g11071
AALT_g11072
AALT_g11073
AALT_g11074
AALT_g11075
AALT_g11076
AALT_g11077
AALT_g11078
AALT_g11079
AALT_g11080
AALT_g11081
cluster28_T1PKS (Mellein)
10 kb
20 kb
30 kb
40 kb
AALT_g11736
AALT_g11737
AALT_g11738
AALT_g11739
AALT_g11740
AALT_g11741
AALT_g11742
AALT_g11743
AALT_g11744
AALT_g11745
AALT_g11746
AALT_g11747
AALT_g11748
AALT_g11749
AALT_g11750
AALT_g11751
AALT_g11752
AALT_g11753
AALT_g11754
AALT_g11755
AALT_g11756
AALT_g11757
AALT_g11758
AALT_g11759
cluster29_NRPS (ACT-Toxin I)
0 kb
10 kb
20 kb
30 kb
40 kb
50 kb
60 kb
70 kb
80 kb
90 kb
AALT_g11948
AALT_g11949
AALT_g11950
AALT_g11951
AALT_g11952
AALT_g11953
AALT_g11954
AALT_g11955
AALT_g11956
AALT_g11957
AALT_g11958
AALT_g11959
AALT_g11960
cluster30_T1PKS (Mellein)
10 kb
20 kb
30 kb
	10 kb
Log2FC
> 4
2 ~ 4
1 ~ 2
-1 ~ 1
-2 ~ -1
-4 ~ -2
< -4
